# The *Hmr* and *Lhr* Hybrid Incompatibility Genes Suppress a Broad Range of Heterochromatic Repeats

**DOI:** 10.1371/journal.pgen.1004240

**Published:** 2014-03-20

**Authors:** P. R. V. Satyaki, Tawny N. Cuykendall, Kevin H-C. Wei, Nicholas J. Brideau, Hojoong Kwak, S. Aruna, Patrick M. Ferree, Shuqing Ji, Daniel A. Barbash

**Affiliations:** Department of Molecular Biology and Genetics, Cornell University, Ithaca, New York, United States of America; Fred Hutchinson Cancer Research Center, United States of America

## Abstract

Hybrid incompatibilities (HIs) cause reproductive isolation between species and thus contribute to speciation. Several HI genes encode adaptively evolving proteins that localize to or interact with heterochromatin, suggesting that HIs may result from co-evolution with rapidly evolving heterochromatic DNA. Little is known, however, about the intraspecific function of these HI genes, the specific sequences they interact with, or the evolutionary forces that drive their divergence. The genes *Hmr* and *Lhr* genetically interact to cause hybrid lethality between *Drosophila melanogaster* and *D. simulans*, yet mutations in both genes are viable. Here, we report that *Hmr* and *Lhr* encode proteins that form a heterochromatic complex with Heterochromatin Protein 1 (HP1a). Using RNA-Seq analyses we discovered that *Hmr* and *Lhr* are required to repress transcripts from satellite DNAs and many families of transposable elements (TEs). By comparing *Hmr* and *Lhr* function between *D. melanogaster* and *D. simulans* we identify several satellite DNAs and TEs that are differentially regulated between the species. *Hmr* and *Lhr* mutations also cause massive overexpression of telomeric TEs and significant telomere lengthening. *Hmr* and *Lhr* therefore regulate three types of heterochromatic sequences that are responsible for the significant differences in genome size and structure between *D. melanogaster* and *D. simulans* and have high potential to cause genetic conflicts with host fitness. We further find that many TEs are overexpressed in hybrids but that those specifically mis-expressed in lethal hybrids do not closely correlate with *Hmr* function. Our results therefore argue that adaptive divergence of heterochromatin proteins in response to repetitive DNAs is an important underlying force driving the evolution of hybrid incompatibility genes, but that hybrid lethality likely results from novel epistatic genetic interactions that are distinct to the hybrid background.

## Author Summary

Sister species capable of mating often produce hybrids that are sterile or die during development. This reproductive isolation is caused by incompatibilities between the two sister species' genomes. Some hybrid incompatibilities involve genes that encode rapidly evolving proteins that localize to heterochromatin. Heterochromatin is largely made up of highly repetitive transposable elements and satellite DNAs. It has been hypothesized that rapid changes in heterochromatic DNA drives the changes in these HI genes and thus the evolution of reproductive isolation. In support of this model, we show that two rapidly evolving HI proteins, Lhr and Hmr, which reproductively isolate the fruit fly sister species *D. melanogaster* and *D. simulans*, repress transposable elements and satellite DNAs. These proteins also help regulate the length of the atypical Drosophila telomeres, which are themselves made of domesticated transposable elements. Our data suggest that these proteins are part of the adaptive machinery that allows the host to respond to changes and increases in heterochromatin and to maintain the activity of genes located within or adjacent to heterochromatin.

## Introduction

As populations diverge, their ability to reproduce with each other diminishes. Hybrid incompatibility (HI), the reduced viability and fertility of interspecific hybrids, is a major cause of reproductive isolation between nascent species and thus an important contributor to speciation. Many of the genes causing HI show evidence of adaptive evolution, typically manifest as excessive numbers of amino-acid-changing mutations compared to neutral expectations [1], [2]. These data do not, however, imply that natural selection acts directly on HI phenotypes. Rather, the prevailing model of HI formulated by Dobzhansky and Muller (D-M) emphasizes that incompatibilities evolve in two distinct steps. First, two or more loci diverge independently in two nascent species. Then, if these species later interbreed, these diverged genes may interact to cause deleterious HI phenotypes. The key insight of the D-M model is that hybrid lethality and sterility evolve as byproducts of intraspecific divergence [1].

Adaptive evolution therefore does ultimately lead to HI, but if we wish to identify the evolutionary forces that drive the divergence of HI genes, then we need to understand the function of these genes within species. The mechanisms by which HI genes cause sterility or lethality are important but separate issues. In fact, it remains uncertain whether the wild type functions of HI genes are generally predictive of the deleterious phenotypes that they cause within hybrids.

Pinpointing the function of HI genes and the causes of their adaptive evolution is a challenging goal. For example, the *Hybrid male rescue* (*Hmr*) gene causes large reductions in hybrid fitness [3]. Loss-of-function mutations in *D. melanogaster*, however, have only moderate effects on fertility and provide few insights into mechanistic underpinnings [4]. The nucleoporins provide an intriguing counterexample. Several have been implicated in hybrid lethality and found to evolve under adaptive evolution [5]. Mutations in nucleoporin subunits are lethal in *D. melanogaster*, but the genes have many pleiotropic functions and the challenge is to pinpoint which one(s) are driving evolutionary divergence.

Here we investigate two hybrid lethality genes, *Lethal hybrid rescue* (*Lhr*) and *Hmr*, which interact to cause F1 hybrid male lethality between *D. melanogaster* and *D. simulans* [6]. Both genes show extensive divergence in their coding sequences that is consistent with positive selection [6], [7]. For *Hmr* this sequence divergence appears to be required for hybrid lethality because the *D. melanogaster* ortholog of *Hmr* causes hybrid lethality but the *D. simulans* ortholog does not [7]. For *Lhr*, however, both orthologs have hybrid lethal activity, with *D. simulans Lhr* having greater activity due to its higher expression level in hybrids [8]. That study left open the possibility that *Lhr* coding sequence divergence makes some contribution to hybrid lethality. Furthermore we found that *Lhr* from the more diverged species *D. virilis* has no hybrid lethal activity, suggesting that more extensive coding sequence divergence does have substantial functional consequences [9].

These previous studies leave unanswered the fundamental question of what evolutionary force is driving adaptive sequence change, and necessitate a detailed understanding of *Hmr* and *Lhr* function within each of the hybridizing species. Loss of function alleles of *Hmr* and *Lhr* are strong suppressors of hybrid lethality, but are largely viable within *D. melanogaster* and *D. simulans*, respectively [10], [11].

Lhr (also known as HP3) protein localizes to heterochromatin [6], [12]. Several other Drosophila HIs also involve heterochromatin or heterochromatin proteins, which is intriguing because genome size varies widely among Drosophila, largely as a consequence of variation in repetitive DNAs that make up the heterochromatin [13], [14]. Heterochromatin may have a much wider role in incompatibility because repetitive DNA variation is the major cause of the ∼1000-fold variation in genome size among multi-cellular eukaryotes [15]. These DNAs can increase in copy number by general host processes such as unequal crossing over and duplication [16]. Alternatively, they may increase copy number by selfish properties such as transposition for TEs [17] and meiotic drive for satellite DNAs [18]. In either case, over-proliferation can be deleterious to their host species by causing genome instability, leading to the evolution of host defense mechanisms [19]. For example, one major mechanism is the piRNA pathway, where small (23–30 nt) RNAs derived from TE sequences are used to silence TE activity [20]. There are also hints that the piRNA pathway may regulate satellite DNAs [21]. Interestingly, piRNA regulatory genes often show signatures of adaptive evolution among Drosophila species [22].

Genetic conflicts with selfish DNAs have been proposed as an important driver of HI [1], [2], [23], but little is known about what specific sequences are interacting with HI genes. *D. simulans* and *D. melanogaster* have great potential for addressing this question because they differ substantially from each other in genome size [14], satellite DNA content [13], [14], and in both the types and number of TEs that they harbor [24]. Here we report that *Hmr* and *Lhr* are required to repress transcription from both TEs and satellite DNAs. *Hmr* and *Lhr* also regulate telomeres, a third specialized type of heterochromatic sequence that serves to protect the ends of linear chromosomes [25] and is composed of rapidly evolving DNA and proteins [26]–[28]. Telomere variation can affect host fitness and genome stability, and has been proposed as another potential source of meiotic drive [27], [29]. We used a *D. simulans* mutation in *Lhr*, comparative cytology, and interspecific complementation with *Hmr* transgenes to identify classes of TEs and satellites that are regulated differentially between the species. We conclude that *Hmr* and *Lhr* provide an adaptive defense against multiple classes of repetitive DNA sequences that change rapidly in evolutionary time, can reduce host fitness, and have high potential to provoke genetic conflict.

## Results

### Lhr and Hmr form a complex with HP1a

**Figure 1:**
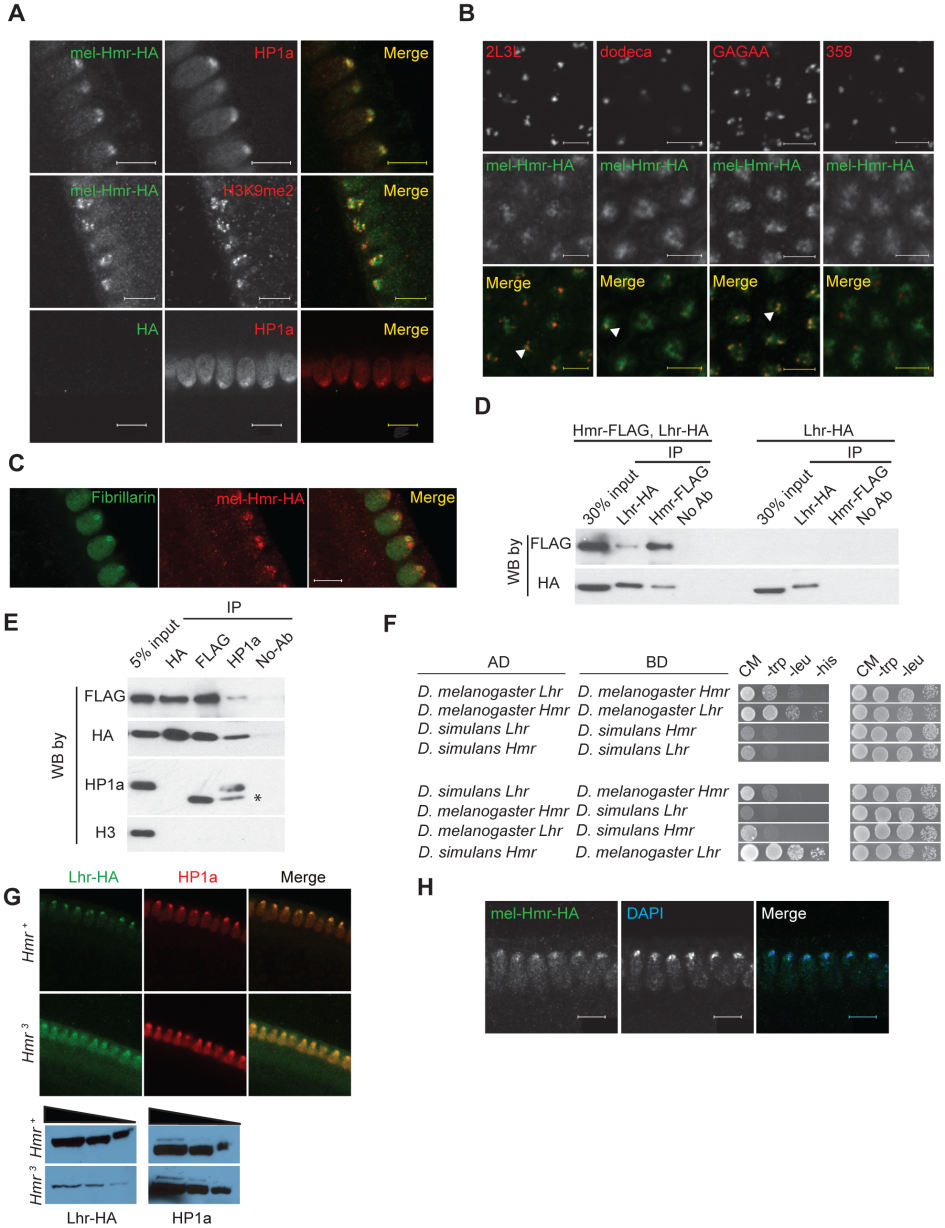
Hmr forms a complex with Lhr and HP1a and is required to stabilize Lhr. (A) mel-Hmr-HA (green) colocalizes with HP1a (top) and H3k9me2 (middle; both red) in nuclear cycle 14 embryos. The HP1a costain is in a *mel-Hmr-HA* background, while the H3k9me2 costain is in a *Hmr^3^; mel-Hmr-HA* background. A negative control shows no HA signal in *w^1118^* embryos lacking the *mel-Hmr-HA* transgene (bottom). Scale bars represent 10 µm. (B) mel-Hmr-HA (green) colocalizes with 2L3L, dodeca and GA-rich satellites but not with the 359 bp repeat satellite in *mel-Hmr-HA* (all FISH probes red). Scale bars represent 5 µm. (C) mel-Hmr-HA (red) colocalizes with the nucleolar marker Fibrillarin (green) in *mel-Hmr-HA* early embryos. Scale bars represent 10 µm. (D) mel-Lhr-HA and mel-Hmr-FLAG co-immunoprecipitate from *D. melanogaster* embryo extracts derived from flies expressing both transgenes (left 4 lanes) but not from flies expressing only Lhr-HA (right 4 lanes). Extracts were IP'd with the indicated antibodies, and then probed by Western Blots (WB) with the same or different antibodies. (E) Lhr-HA, Hmr-FLAG and HP1a co-immunoprecipitation from embryo extracts. Specificity is indicated by lack of immunoprecipitation of histone H3. Asterisk indicates the antibody light chain. (F) Lhr and Hmr interact in a yeast-two hybrid assay. Interactions were detected by growth on complete media (CM) lacking histidine (his); growth controls were performed on CM lacking tryptophan (trp) and leucine (leu). The top 4 panels test for interactions between orthologs from the same species; the bottom 4 between heterospecific orthologs. AD, activation domain; BD, DNA binding domain. (G) Lhr-HA is detectable in *Hmr^3^* and localizes to heterochromatin, as indicated by co-localization with HP1a. Note that a higher gain was used in the *Hmr^3^* panels compared to the *Hmr^+^* panels in order to detect Lhr-HA, and is reflected in the higher background. Western blots confirm that Lhr-HA levels are reduced in *Hmr^3^*. HP1a is used as a loading control. (H) Hmr-HA maintains its localization to DAPI-dense heterochromatin in *Lhr^KO^; Hmr-HA* embryos. Scale bars represent 10 µm.

Lhr protein localizes to a subdomain of pericentric heterochromatin in early embryos [8]. To explore possible similarities with Hmr, we examined the localization of Hmr with a 3X-HA epitope-tagged *Hmr* transgene (see Materials and Methods). mel-Hmr-HA colocalizes with HP1a and H3K9me2 at heterochromatin in nuclear cycle 14 embryos (Figure 1A). We then used Immuno-FISH to determine its localization relative to specific heterochromatic satellite DNA sequences. mel-Hmr-HA does not overlap with the X-linked 359-bp satellite but colocalizes with dodeca, a GC-rich pericentromeric satellite on chromosome 3. This pattern mimics that seen previously with Lhr [8]. Additionally, mel-Hmr-HA colocalizes with GA-rich repeats and the 2L3L satellite in embryos (Figure 1B). Colocalization between mel-Hmr-HA with both dodeca and GA-rich repeats is also observed in ovarian nurse cells from *Hmr^3^; mel-Hmr-HA* females, indicating that localization is not a consequence of overexpression (Figures S1B, C). Unlike Lhr [8], mel-Hmr-HA localizes to the nucleolus in early embryos (Figure 1C), suggesting that Hmr may have some functions distinct from Lhr.

The largely similar localization patterns of Hmr and Lhr raise the possibility that they physically interact. We performed co-immunoprecipitation (co-IP) studies from embryo extracts and found that mel-Lhr-HA and mel-Hmr-FLAG co-IP (Figure 1D). *mel-Lhr-HA* was previously shown to express at wild type levels [8], and *mel-Hmr-FLAG* is expressed significantly lower than wild type levels (Figure S2), demonstrating that these results are not due to overexpression. Lhr was previously shown to bind to, co-localize with, and be dependent on HP1a for correct heterochromatic localization [6], [9], [12], [30]. We therefore tested if HP1a also associates with Hmr. IPs with HP1a pulled down mel-Lhr-HA and mel-Hmr-FLAG, but the reciprocal IPs failed to pull down detectable HP1a (Figure 1E).

Yeast two-hybrid assays show that *Hmr* and *Lhr* from *D. melanogaster* interact, suggesting that the co-IP reflects a direct interaction between the proteins (Figure 1F). This interaction is likely mediated via the BESS domains within Lhr and Hmr [6], a 40 amino-acid motif found in 19 proteins in *D. melanogaster* that has been implicated in protein-protein interactions and homo-oligomerization [31]. We also found that the *D. simulans* orthologs interact, as do the heterospecific combinations; the strength of interactions varied widely but exploring the potential significance of this result will require a more quantitative assay.

We next examined protein localization in mutant backgrounds to test the potential mutual dependence of Lhr and Hmr for their localization to heterochromatin. We made a *D. melanogaster Lhr* mutation by recombining a *mini*-*white* gene into the *Lhr* locus to create the *Lhr^KO^* allele (Figure S3A). In *Lhr^KO^*, transcription from *Lhr* but not flanking genes is greatly reduced, and no Lhr protein is detectable (Figure S3B, C). These results demonstrate that *Lhr^KO^* is a strong loss of function allele, which we confirmed in hybrid rescue crosses (see Materials and Methods).

Lhr-HA levels are greatly reduced in *Hmr^3^* mutant embryos but when examined at high gain a small amount of Lhr-HA is detectable in heterochromatin (Figure 1G). This result suggests that Hmr is not absolutely required to localize Lhr to heterochromatin, though it remains possible that some Hmr protein is made in the *Hmr^3^* mutant. In a reciprocal experiment, Hmr-HA localization appears normal in *Lhr^KO^* (Figure 1H). In combination with previous results, our data suggest that Lhr localization to heterochromatin depends on HP1a, and that Hmr stabilizes Lhr.

### *Lhr* is required for female fertility

**Figure 2:**
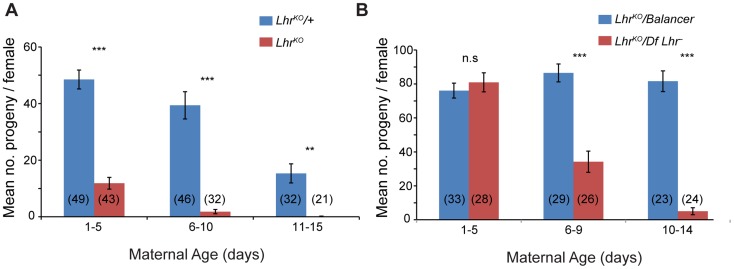
*Lhr* mutant females have reduced fertility. Total adult progeny from single *Lhr^KO^/Lhr^KO^* (A) or *Lhr^KO^*/*Df(2R)BSC44*, *Lhr^−^* (B) females were compared at 27° to heterozygous female siblings (*Lhr^KO^/+* for (A); *Lhr^KO^/SM6a* for (B)). The difference between the fertility of genotypes was tested by a two-tailed *t*-test. n.s = not significant, **p<0.01,***p<0.001. The number of individuals tested for each experiment is shown at the bottom of the bars. The error bars represent S.E.M. Crosses were performed at 27°.

*Lhr^KO^* flies are almost fully viable (22.25% compared to the expected 25% in crosses between heterozygotes at 27°; *p*<0.05 by Chi-squared; N = 2813 total flies scored). However, comparison of *Lhr^KO^* with a background-matched *Lhr^+^* control (see Materials and Methods) showed that *Lhr^KO^* females have substantially lower fertility, particularly at higher temperatures. One to five day old *Lhr^KO^* females display only a fraction of the fertility of *Lhr^KO^/+* and later become sterile (Figure 2A). We confirmed this in a different *Lhr^−^* background where a similar reduction in fertility occurs at later ages (Figure 2B). In a separate experiment we found that the hatch rate of the eggs laid by *Lhr^KO^/Lhr^KO^* mothers is low and declines with increasing maternal age (Table S1). This *Lhr^KO^* female fertility phenotype is strikingly similar to that of *Hmr* mutants [4], suggesting that *Hmr* and *Lhr* may function in a common regulatory pathway.

### *Lhr* and *Hmr* are required to repress transposable elements

**Figure 3:**
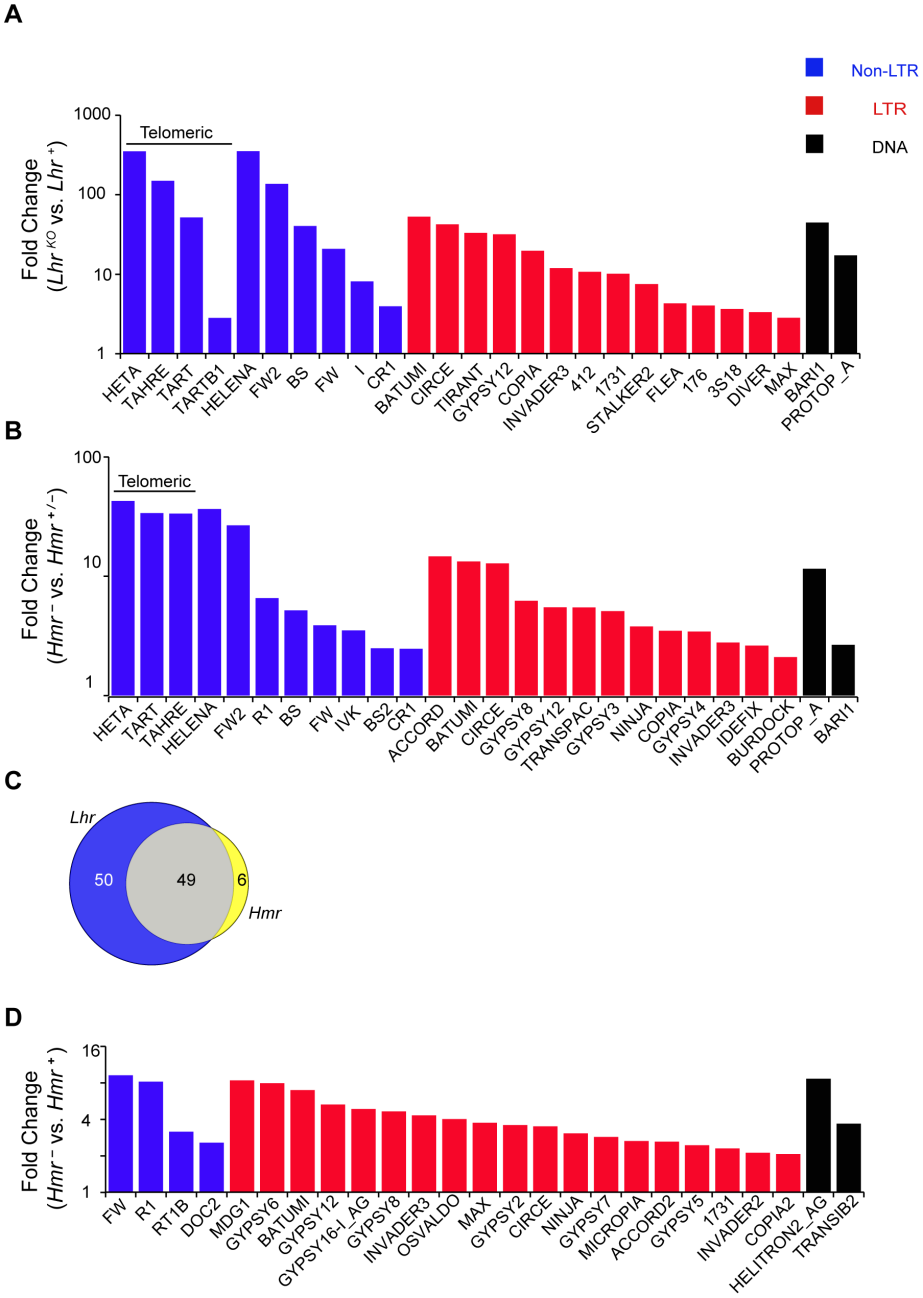
TE misregulation in *Lhr* and *Hmr* mutants. (A and B) Analysis of *Lhr^KO^* (A) and *Hmr^−^* (B) ovaries. Reads with zero mismatches were mapped separately to the individual-insertion or consensus-sequence TE databases. A subset of TEs that are significantly different between genotypes are shown and include those with the 25 lowest p-values obtained from individual-insertion mapping analysis, but excluding all *centroid* repeats [98]. Additionally shown are *TAHRE*, which is only found in the consensus-sequence database, as well as *TARTB1* for *Lhr^KO^*, which is significant but not among the 25 top hits in the *Lhr^KO^* individual-insertion analysis. (C) 49 TEs are upregulated at least 2 fold in both *Lhr^KO^* and *Hmr^−^*. TE families include those resulting from mapping reads to the insertion database, as well as families found only when reads were mapped to the consensus database. (D) Reads from *Hmr* mutant or wildtype male larvae with up to three mismatches were mapped to the individual-insertion or consensus-sequence TE databases. All TE families, excluding *centroids*, that were significantly upregulated in the insertion sequence based analysis are shown here. Note the different Y-axis scales in A, B and D. Classification of DNA, LTR and non-LTR elements is from reference [99].

We performed an RNA-Seq comparison of ovaries from *Lhr^KO^* and *Lhr^+^* to investigate the cause of this fertility reduction and discovered a widespread increase in transposable element (TE) transcripts. Using two different TE mapping methods (see Materials and Methods) we found that transcripts from 99 families were at least 2-fold upregulated, with 38 elements being at least 10 fold upregulated (Figure 3A; Table S2). Mis-regulated TEs include elements with germline expression such as the telomeric non-LTR retrotransposons *HeT-A* (350.7 fold) and *TART* (51.76 fold), the LTR retrotransposon *copia* (19.8 fold), and the DNA transposon *bari-1* (44.7 fold). TEs expressed only in the somatic follicle cells, such as *Gypsy* (3.8 fold) and *Zam* (7 fold) were also upregulated. In addition, qRT-PCR in two different genetic backgrounds confirmed the massive increase in *HeT-A* transcript levels (185–846-fold; Figure S4). These results demonstrate that the telomeric TEs are especially sensitive to *Lhr* regulation.

We also performed RNA-Seq analysis of an *Hmr* mutant (*Df(1)Hmr^−^/Hmr^3^*, abbreviated below as *Hmr^−^*). We compared it to a heterozygous control (*Df(1)Hmr^−^/y w Hmr^+^*, abbreviated below as *Hmr^−^*/*Hmr^+^*) because it closely matches the genetic background of the mutant genotype, and also serves as a control for *Hmr* transgenic genotypes that are described below. We found that 55 different TE families are upregulated at least 2 fold in *Hmr* mutants, with 14 being upregulated at least 10 fold (Figure 3B; Table S3). Notably, the telomeric retrotransposons *HeT-A* and *TART* are again among the most highly upregulated. Strikingly, the TEs affected by *Hmr* are largely a subset of *Lhr*-regulated TEs, suggesting that they act together to regulate multiple TE families (Figure 3C). The smaller number of mis-regulated families in *Hmr^−^* likely reflects the fact that we are comparing *Hmr^−^* mutants to heterozygotes, but *Lhr* mutants to wild type.

Since some germline TE repressor genes also regulate somatic TE expression [32], we performed RNA-Seq to compare TE expression between 72–76 hour-old *Df(1)Hmr^−^/Y* and *Hmr^+^/Y D. melanogaster* male larvae. This also served as a control for experiments described below to address whether TE mis-expression may be contributing to hybrid lethality. We found that 31 TEs exhibit a statistically significant ≥2 fold upregulation (Figure 3D; Table S4), but there are two striking differences compared to *Hmr* mutant ovaries. First, different TEs are affected, with the telomeric retrotransposons in particular not upregulated in the larvae. Second, the magnitude of TE derepression is lower in larvae.

### *Lhr* and *Hmr* affect expression of heterochromatic genes

**Figure 4:**
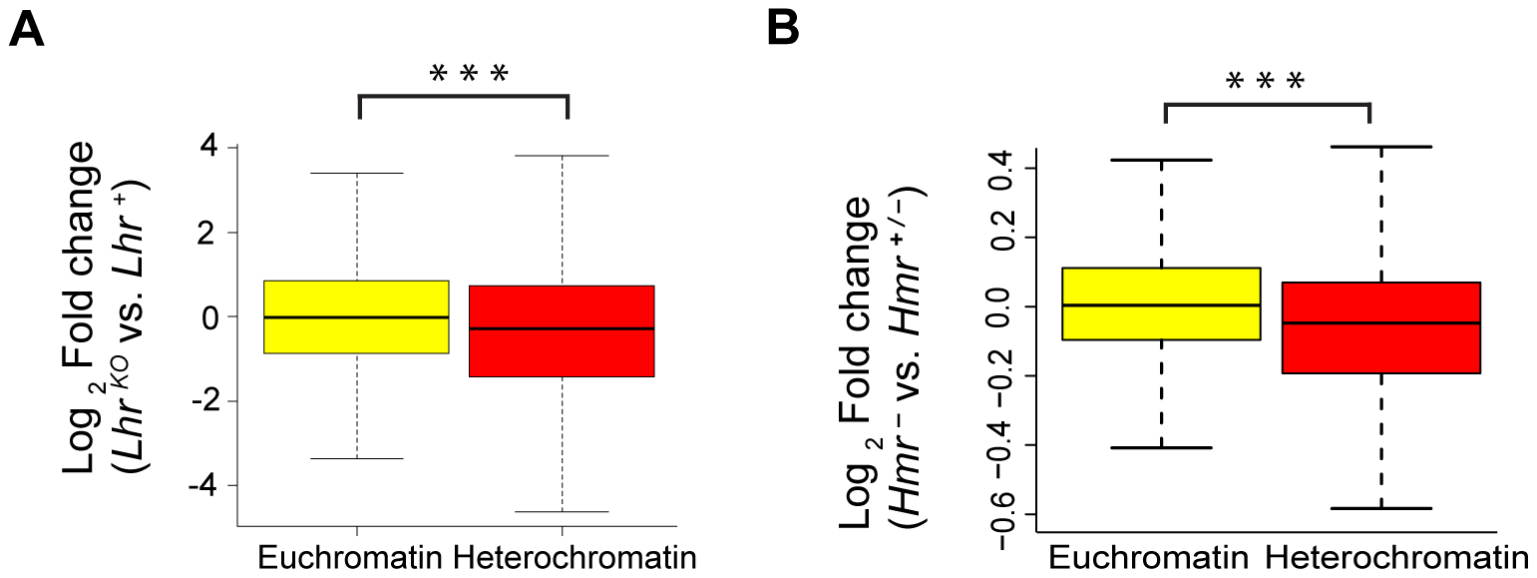
Reduced expression of heterochromatic genes in *Lhr* and *Hmr* mutants. Loss of *Lhr* (A) and *Hmr* (B) leads to a statistically significant reduction in the expression of heterochromatic genes. Significance of difference was calculated using the Wilcoxon rank sum test with continuity correction (for (A) *p* = 3.549e-05, for (B) *p* = 1.461e-09). Box plots show log_2_ fold change of 7838 euchromatic and 370 heterochromatic genes for (A) and 7451 euchromatic and 344 heterochromatic genes for (B). The definition of the euchromatin-heterochromatin boundary for all chromosomes comes from experiments done in S2 tissue culture cells, except for 3R, which comes from the cytogenomic border [100].

We next examined potential effects on protein-coding genes. Remarkably few genes (11 in *Hmr^−^*; 0 in *Lhr^KO^*) show a statistically significant misregulation in either *Lhr* or *Hmr* mutants (FDR 0.05; Tables S5, S6). However, a comparison of fold change in the expression of all heterochromatic versus all euchromatic genes found that heterochromatic genes are downregulated to a greater extent for both mutants, although the effect is stronger in *Lhr^KO^* (Figure 4). Lhr preferentially associates with heterochromatic genes in an embryonic cell culture line [12]; our results suggest that Lhr and Hmr have a small positive effect on expression of some heterochromatic genes.

### *Lhr* and *Hmr* mutants have long telomeres

**Figure 5:**
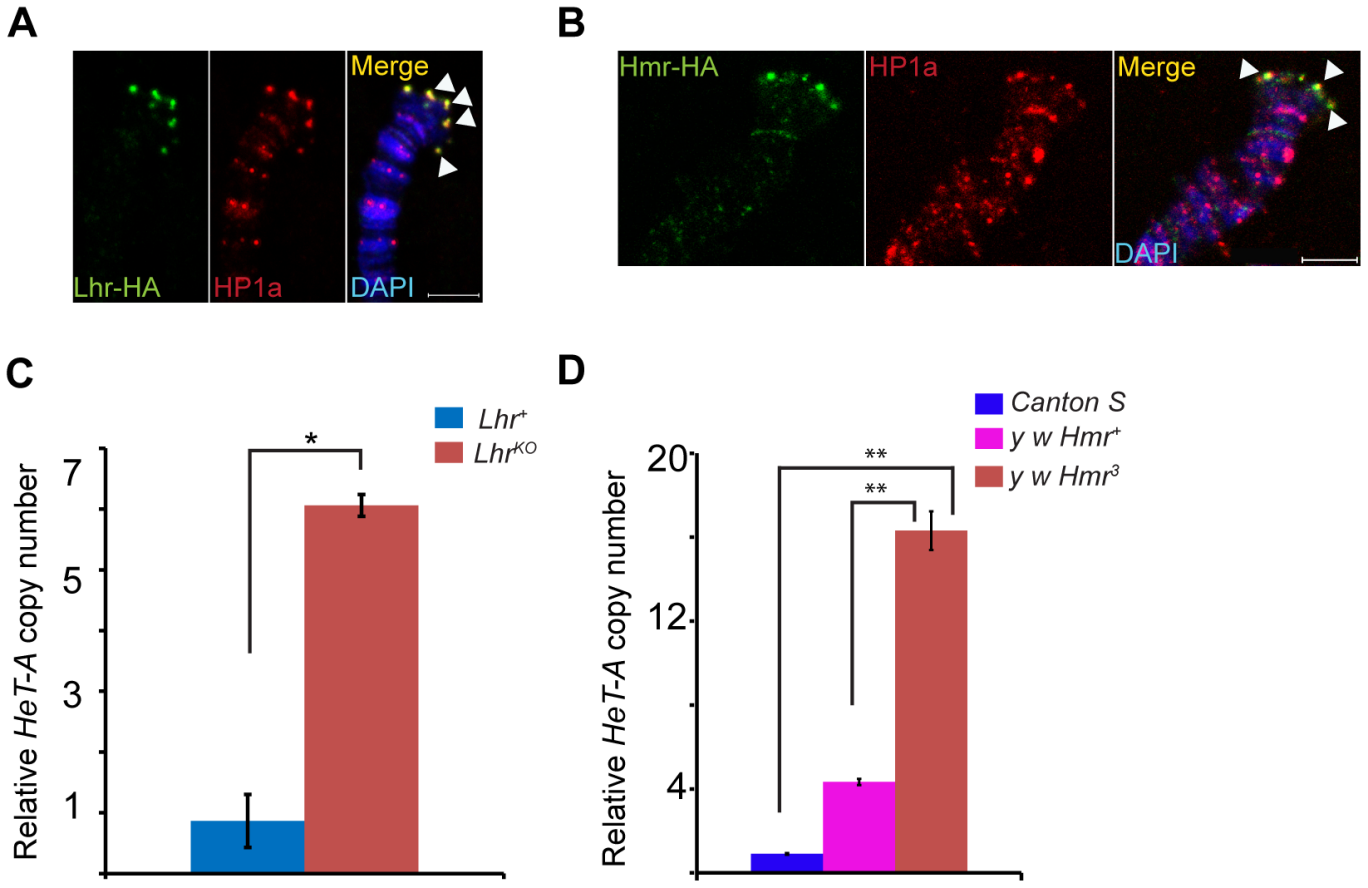
Lhr and Hmr are telomere cap proteins required for regulating telomere length. Lhr-HA (A) and Hmr-HA (B) localize to telomeres. Co-immunostaining with anti-HA and anti-HP1a shows that both proteins colocalize at the cap (arrowheads). The merged images include DAPI to stain DNA, shown in blue. *Lhr^KO^* (C) and *Hmr^3^* (D) have increased *HeT-A* copy number. qPCR was used to estimate the abundance of *HeT-A* and *rp49* from *Lhr^KO^*, *Lhr^+^*, *y w Hmr^3^*, a matched *y w Hmr^+^* control, and the wild-type Canton S strain. Genomic DNA was isolated from carcasses of females whose ovaries were removed in order to minimize the amount of polytenized DNA present. Relative *Het-A* copy number is the ratio of *Het-A/rp49*. The error bars represent S.E.M for three replicates. The significance of the differences between the genotypes was calculated using two tailed *t*-test; * = *p*<0.05; ** = *p*<0.01. Scale bars = 5 µm.

Drosophilidae have lost the telomerase-based mechanism of telomere elongation and instead use the regulated transposition of the *HeT-A*, *TART* and *TAHRE* retrotransposons [33]. Strikingly, these were among the 3 most strongly affected TEs in *Lhr^KO^* and *Hmr^−^* ovaries (Figure 3). We therefore investigated in more detail the localization of Lhr and Hmr proteins to the telomere [6]. Cytological markers on polytene chromosomes have been used to describe three distinct regions in the telomere, with HP1a localizing exclusively to the “cap”, a proteinacous structure at the most distal end of telomeres [25], [28].

mel-Lhr-HA and mel-Hmr-HA overlap with HP1a, showing that Lhr and Hmr localize to the cap but not to more proximal regions (Figure 5A, B). Localization is not due to the doubling of the dosage of these proteins in the transgenic lines because it also occurs in the *Hmr^3^; Hmr-HA/Hmr-HA* and *Lhr^KO^/+; Lhr-HA/+* genotypes (Figure S5). The localization of Lhr and Hmr to the cap, the primacy of the cap in the regulation of telomeric length, and the increase in the transcript levels of telomeric retro-transposons in *Lhr* and *Hmr* mutants led us to ask if these mutations cause long telomeres. We quantitated *HeT-A* DNA copy number by qPCR in *Lhr^KO^* flies maintained at 27°C separately from its matched wild-type control strain for ∼40 generations. We found that *HeT-A* copy number increased approximately 6 fold in *Lhr^KO^* (Figure 5C). We also examined *HeT-A* DNA copy number in an *Hmr^3^* mutant stock, and found ∼4–16 fold higher abundance than in the *Hmr^+^* stocks *y w* and Canton-S (Figure 5D).

### Satellite DNA transcripts are upregulated in *Lhr* and *Hmr* mutants

**Figure 6:**
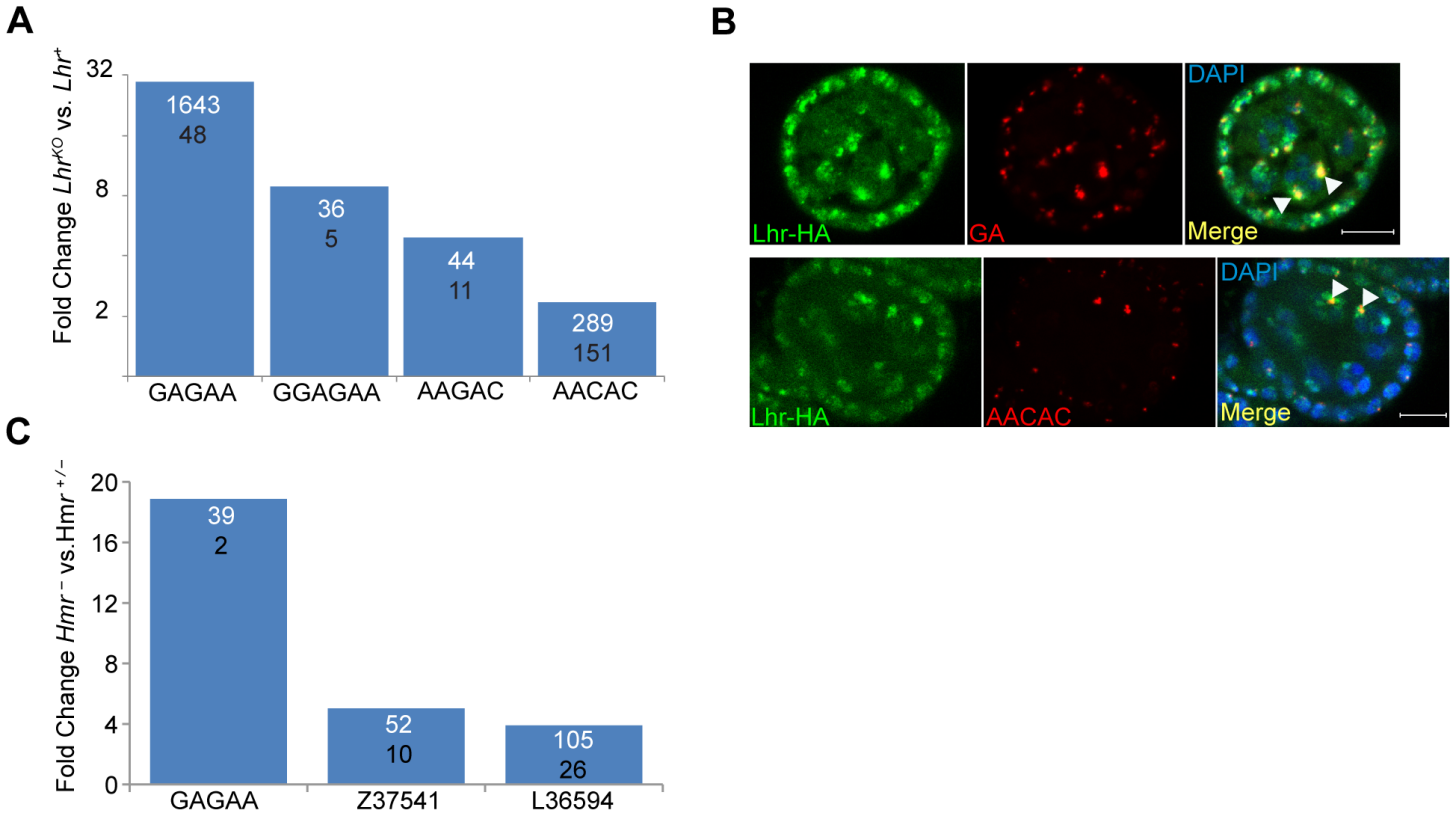
*Lhr* and *Hmr* repress satellite DNA transcription. (A) Fold increase in satellite transcripts of *Lhr^KO^* versus *Lhr^+^*. Numbers within the bars show normalized reads mapping to each satellite, the numerator from *Lhr^KO^* and the denominator from *Lhr^+^*. All differences have *p*<0.01 by F.E.T. test. (B) Lhr-HA (green) colocalizes with GA-rich and AACAC satellites (red) in ovarian nurse cell nuclei (arrowheads). DAPI is shown in the merged images in blue. Scale bar = 10 µm. (C) Fold increase in satellite transcripts in *Hmr^−^* versus *Hmr^+/−^*. Numbers within the bars show normalized reads mapping to each satellite, the numerator from *Hmr^−^* and the denominator from *Hmr^+/−^*. All differences have *p*<0.001 by F.E.T. test.

Hmr and Lhr both localize to pericentric heterochromatin, which is largely composed of TEs and satellite DNAs. The potential effects of heterochromatin proteins on the levels of transcripts from satellites have not been widely explored. We therefore used our RNA-Seq data to examine transcript levels from 143 repeats in a repeat-sequence database (see Materials and Methods). Transcripts from most repeats are found at low abundance in *Lhr^+^* with only 17 producing more than 10 reads (Table S7). Four different satellite classes are significantly higher in *Lhr^KO^* versus *Lhr^+^* ovaries, including three that collectively make up more than 8% of the *D. melanogaster* genome [13]: AAGAC, AACAC, and the GA-rich satellites (Figure 6a). The GAGAA satellite showed the strongest effect, with an approximately 30-fold increase.

These results raise the question of whether transcriptional regulation of specific satellite DNAs reflects a direct association with Lhr. Lhr was not previously tested for association with either GA-rich satellites, which are found on all chromosomes in *D. melanogaster* [34], or with the AACAC satellite found on chromosomes 2 and Y [35]. We found that Lhr-HA colocalizes extensively with the GA-rich and AACAC satellites in the nurse cell nuclei of early stage egg chambers (Figure 6B, S1A).

In our *Hmr* RNA-Seq data the number of reads mapping to each repeat family was generally very small, but 3 satellite families are significantly derepressed by at least 4 fold in *Hmr^−^* (Figure 6C; Table S8), including GAGAA, which has a 19 fold increase in expression. This finding is consistent with the localization of mel-Hmr-HA to GA-rich satellites above (Figure 1B). Additionally, the satellite Z37541, which binds nuclear lamins, is upregulated 5 fold in *Hmr^−^* [36].

Although Lhr-HA localizes to the dodeca satellite [8]; we detected very few reads in either our *Lhr^+^* or *Lhr^KO^* samples; likewise we did not find upregulation of dodeca in our *Hmr* RNA-Seq data. We conclude that Hmr and Lhr proteins are required to regulate transcript levels of a subset of satellites to which they localize.

### siRNA and piRNA patterns are largely normal in *Lhr^KO^*

**Figure 7:**
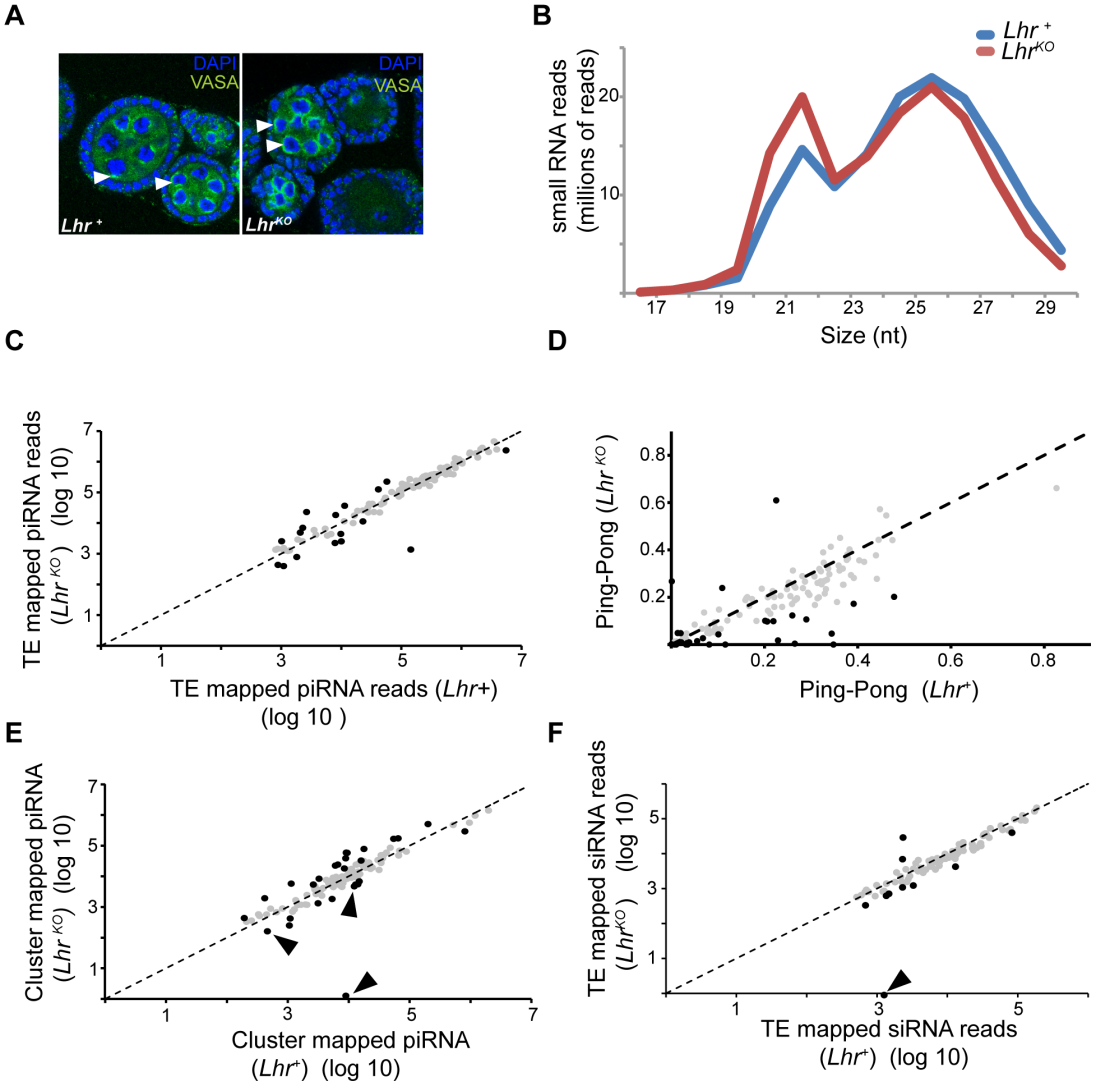
Small RNA patterns are largely unaffected in *Lhr^KO^*. (A) VASA (green) marks the peri-nuclear nuage (white arrowheads) and shows no difference in localization between *Lhr^+^* and *Lhr^KO^* ovaries. (B) siRNA (17–22 nt) without mismatches and piRNA (23–30 nt) with up to one mismatch were mapped to a reference sequence set containing the *D. melanogaster* r5.68 genome, *D. melanogaster* sequences from Repbase and the repeat-sequence database. The number of mapped *Lhr^KO^* reads was normalized to the total number of mapped *Lhr^+^* reads. (C) Filtered piRNA reads were mapped uniquely to the Repbase TE consensus sequences with one allowed mismatch. 121 TE families producing > = 1000 reads summed over both genotypes are shown. Black circles represent TE families whose fold change between *Lhr^KO^* and *Lhr^+^* is greater than 2 fold (*p<0.001*). (D) Ping-pong scores of TE families in *Lhr^KO^* and *Lhr^+^*. Black circles represent TE families whose fold change in ping-pong score between *Lhr^KO^* and *Lhr^+^* is greater than 2 fold (Table S10). (E) Plot shows the number of unique piRNAs mapped to piRNA clusters, with one allowed mismatch and normalized between genotypes. piRNA clusters with > = 500 reads summed over both genotype are shown. Black arrowheads point to sub-telomeric piRNA clusters. Black circles indicate clusters whose fold change between *Lhr^KO^* and *Lhr^+^* is greater than 2-fold (*p<0.001*). (F) Unique siRNA (17–22 nt) were mapped as in (C), except no mismatches were allowed. 96 TE families are plotted that have > = 1000 reads summed over both genotypes. Black circles represent TEs whose siRNA levels changed by >2 fold. siRNA mapping to the TAS repeat HETRP are almost completely lost (arrow). For (C, D, F) significance values were calculated using F.E.T., implemented in DEG-seq.

The wide spectrum of TEs derepressed in *Lhr* and *Hmr* mutants is similar to mutations in piRNA regulatory genes such as *Ago3* and *aub* that post-transcriptionally regulate TEs via small-RNA-mediated silencing [37], [38]. We therefore investigated a range of phenotypes that are associated with defects in the piRNA pathway. *Ago3* and *aub* mutants disrupt Vasa localization to the peri-nuclear small-RNA processing center, the nuage, and exhibit drastic reductions in the piRNA fraction (23–30 nt) [38], [39]. We found, however, that Vasa localizes normally in *Lhr^KO^* (Figure 7A). We then sequenced the small RNA pool in *Lhr^KO^* and found that the piRNA level is broadly comparable to *Lhr^+^* with only a minor reduction in longer piRNAs (Figure 7B). This pattern contrasts with mutants such as *aub* and *spn-E* that show a severe loss of piRNAs [39]. We looked more closely for TE-specific defects and found that piRNAs mapping to most individual TE families are comparable between *Lhr^+^* and *Lhr^KO^* (Figure 7C; Table S9). We also examined “ping-pong” processing, which produces piRNAs from opposing strands with a characteristic 10 nucleotide overlap [38], [39]. Ping-pong scores are generally higher in *Lhr^+^* (Figure 7D; Table S10) but several points argue against there being a significant defect in ping-pong or piRNA processing in *Lhr^KO^*. First, the magnitude of the difference between genotypes is low, with the ping-pong score being > = 2-fold higher in *Lhr^+^* for only 26/140 TEs. Furthermore, half of these 26 have ping-pong scores <0.10 in *Lhr^+^* (Table S10), suggesting that those TE families are not significantly processed by ping-pong in wild type flies. Second, these differences in ping-pong scores between *Lhr^+^* and *Lhr^KO^* are much milder compared to mutations in genes such as *spn-E* [39]. Third, many of the TEs showing differences in ping-pong scores are not strongly depressed in *Lhr^KO^*. Conversely, many TEs that are strongly derepressed in *Lhr^KO^*, including *HeT-A*, have ping-pong scores that are comparable to wild-type. Fourth, some TEs with elevated mRNA levels also show increased ping-pong signatures, probably because of increased processing through a functional ping-pong pathway. We suggest therefore that the moderate trend towards reduced ping-pong scores in *Lhr^KO^* does not reflect a failure in the ping-pong cycle. Instead, it may result from a skew in the ratio of sense∶antisense piRNAs, because *Lhr^KO^* flies have high levels of TE transcripts that can be processed into sense piRNAs. An analogous argument has been made for mutations in the Drosophila *Gtsf1/asterix* gene, which derepress TEs and give an altered ratio of sense and antisense piRNAs but appear to do so downstream of piRNA biogenesis [40].

We searched further for possible defects in piRNA production by examining piRNAs that map to 122 primary-piRNA-generating heterochromatic clusters [41]. piRNAs originating from most of the major clusters are not significantly affected in *Lhr^KO^* but 16 and 11 of the 122 clusters are at least two-fold higher or lower, respectively, in *Lhr^KO^* (Figure 7E; Table S11). Some of the most strongly affected clusters are associated with telomeres. Cluster 3 consists entirely of telomeric retrotransposons and is upregulated 4.3 fold in *Lhr^KO^*. Sub-telomeric cluster 11 shows a complete loss of unique piRNAs, while clusters 33 and 4 are 2.6 and 2.9 fold downregulated, respectively. These 3 clusters consist mainly of HETRP telomere-associated (TAS) repeats and are therefore not expected to contribute to TE repression; their misregulation instead suggests that *Lhr* is required for regulating chromatin states at telomeres.

The siRNA pathway has also been implicated in repressing TEs in the ovary [42]–[44]. We found that siRNAs mapping to the vast majority of TE families, including those mapping to *HeT-A*, are not significantly different between *Lhr^KO^* and *Lhr^+^*, suggesting that *Lhr* is not generally required for siRNA biogenesis (Figure 7F; Table S12). Taken together, our results indicate that defects in small RNA synthesis are not the cause of TE derepression in *Lhr^KO^*. An intriguing possibility is that Lhr is a piRNA-dependent effector of TE silencing.

### Comparing *Lhr* function in *D. simulans* and *D. melanogaster*

We propose that the dynamic sequence turnover of repetitive DNAs is the selective pressure driving the adaptive sequence divergence of *Lhr* and *Hmr*. This hypothesis implies that the localization and/or function of the Lhr protein have changed between species, due to co-evolution with species-specific repetitive DNAs. The *Lhr^1^* allele in *D. simulans* [10] presents a rare opportunity to compare the function of a rapidly evolving heterochromatin protein between sibling species. We performed RNA-Seq from ovaries of *Lhr^1^* females and a matched *Lhr^+^* control (see Materials and Methods). We found essentially no *Lhr* transcript reads in the *Lhr^1^* mutant strain (Table S13), strongly suggesting that this allele is null.

**Figure 8:**
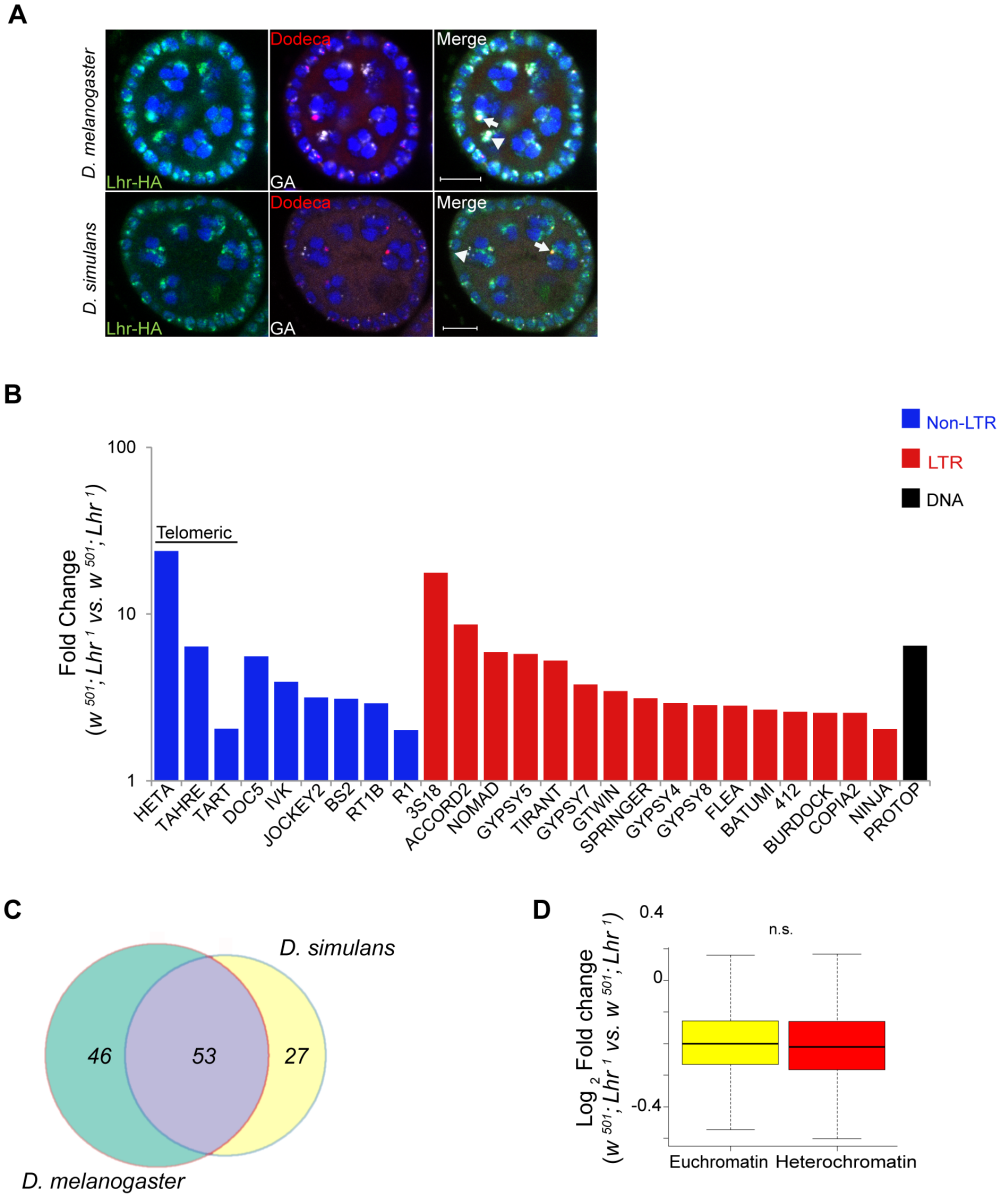
Analysis of *Lhr* function in *D. simulans*. (A) Immuno-FISH experiment shows that the brightest mel-Lhr foci colocalize with dodeca (red, arrow) and GA satellites (white, arrowhead) in *D. melanogaster* (upper panel). The brightest sim-Lhr foci either colocalize or are juxtaposed with dodeca (arrow) but are not associated with GA-rich satellites (arrowhead). All panels contain DAPI shown in blue. Scale bar = 10 µm. (B) Fold changes in TE expression between *w^501^; Lhr^1^* and *w^501^; Lhr^+^* were calculated for uniquely mapping reads with zero mismatches to the individual-insertion database and with three mismatches to the consensus-sequence database. Three mismatches are required to account for the divergence of TE insertions in *D. simulans* from the consensus sequences, which are largely defined from *D. melanogaster* TEs. The 25 most significantly derepressed TE families in the individual-insertion sequence based analysis are shown here (excluding *centroids*), as well as *TAHRE*, which is found only in the consensus-sequence database. Classification of DNA, LTR and non-LTR elements is from reference [99]. (C) Comparison of TE misregulation between *D. melanogaster* and *D. simulans Lhr* mutations. The diagram includes all TE families that were upregulated at least two fold, including those in individual-insertion database analysis as well as those that are only represented in the consensus-sequence database analysis. (D) Comparison of euchromatic and heterochromatic gene expression in *D. simulans w^501^; Lhr^1^*, as described in Figure 4. The euchromatin-heterochromatin border has not been experimentally determined in *D. simulans* and was defined from *D. melanogaster*, Analysis includes 7479 euchromatic and 350 heterochromatic genes (*p* = 0.12, Wilcoxon rank sum test with continuity correction).

*D. simulans* has many of the same satellites as *D. melanogaster* but they are generally of lower abundance [13]. We therefore first examined satellite DNA expression in the *Lhr^1^* and *Lhr^+^* (control) RNA-Seq data. Unlike in *D. melanogaster Lhr^KO^*, we found few satellite reads in either genotype and no significant differences between them. We conclude that *Lhr* has a unique role in *D. melanogaster* to repress satellite DNA transcription. The AACAC satellite that Lhr co-localizes with in *D. melanogaster* (Figure 6B) is absent in *D. simulans* [35]. The GAGAA satellite is also drastically different in *D. simulans*, being eight-fold less abundant and found only on the sex chromosomes [13], [35]. To determine if this interspecific difference in satellite content reflects divergent localization of Lhr orthologs, we examined *D. simulans* ovaries expressing a previously characterized sim-Lhr-HA transgene [8]. While Lhr-HA is juxtaposed to dodeca in both species, as previously described [8], the strongest foci in *D. simulans* do not overlap with GAGAA (Figure 8A). These results demonstrate that Lhr has evolved distinct localization patterns to at least two satellites between *D. melanogaster* and *D. simulans*.

We next examined TE expression and discovered a broad spectrum of TEs derepressed in *D. simulans Lhr^1^*, with 80 TEs showing a greater than two-fold up-regulation (Figure 8B; Table S14). Upregulated TEs again include the telomeric transposable elements *HeT-A*, *TART*, and *TAHRE*, other germline elements such as *Nomad*, and somatic TEs such as *Zam* and *Gypsy 5*. 53 transposable elements were commonly mis-regulated in both *D. melanogaster* and *D. simulans*, showing that the function of *Lhr* in repressing TEs is broadly conserved between species (Figure 8C). However, the fold increases of most individual TE families are lower than seen in *D. melanogaster Lhr^KO^*. For example, *HeT-A* is 352 fold upregulated in *Lhr^KO^* but only 23.8 fold upregulated in *Lhr^1^*.

We further discovered that *Lhr* loss in *D. simulans* does not significantly affect the expression of heterochromatic genes (Figure 8D, Table S13), in contrast with our similar analysis of *Lhr^KO^* in *D. melanogaster* (Figure 4A). This result suggests that pericentric genes in *D. melanogaster* are more sensitive to changes in heterochromatin state than in *D. simulans*. Overall, our results demonstrate that *Lhr* function correlates with the increased repeat content and larger amount of heterochromatin found in *D. melanogaster*.

### Comparison of *Hmr* ortholog function

**Figure 9:**
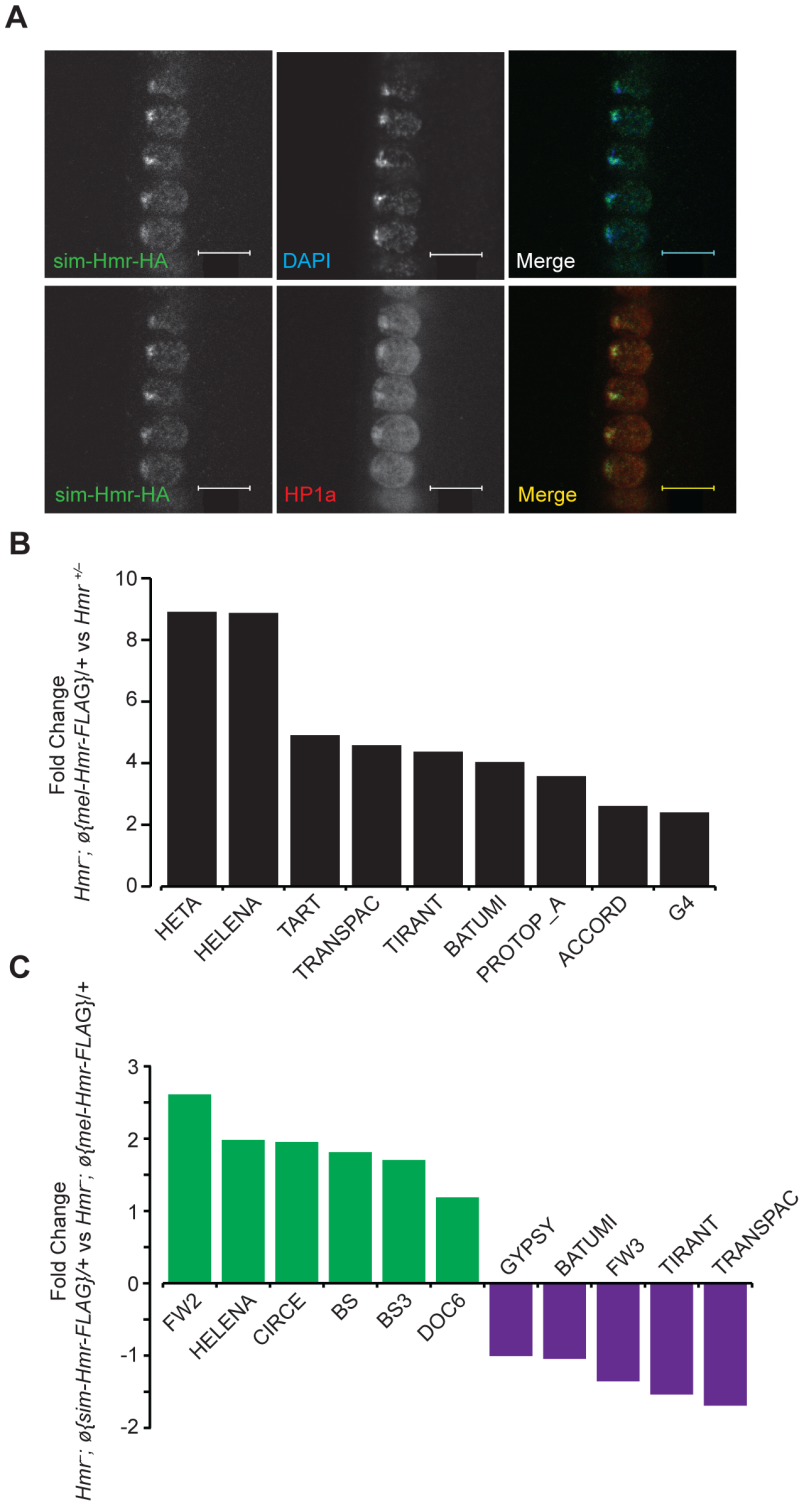
*Hmr* orthologs have diverged in their effects on a small subset of TEs. (A) sim-Hmr-HA colocalizes with HP1a (red) in nuclear cycle 14 *D. melanogaster Hmr^3^*; *sim-Hmr-HA* embryos. The *sim-Hmr-HA* transgene was transformed into *D. melanogaster* at the identical *attP2* site used for *mel-Hmr-HA* above (Figure 1). DAPI is shown in blue. (B) *mel-Hmr-FLAG* does not fully complement TE derepression in *Hmr^−^*. 9 TE families are 2–9× more highly expressed in *Hmr^−^*; *ø{mel-Hmr-FLAG}/+* compared to *Hmr^+/−^*. (C) Comparison of TE expression in *Hmr^−^; ø{mel-Hmr-FLAG}/+* and *Hmr^−^; ø{sim-Hmr-FLAG}/+*. For B and C, reads were mapped to the individual-insertion database. TEs are considered differentially expressed in the pairwise comparisons if there was at least a 2× fold change and *p*<0.001.

To examine the functional consequences of *Hmr* divergence, we took an alternative approach of transforming *sim-Hmr* transgenes into *D. melanogaster*. We found that sim-Hmr-HA, like mel-Hmr-HA, localizes to heterochromatin in *D. melanogaster* (Figure 9A).

To examine potential differences in TE and satellite regulation, we used parallel *mel-Hmr-FLAG* and *sim-Hmr-FLAG* transgenes, crossed them into an *Hmr^−^* background (*Df(1)Hmr^−^/Hmr^3^*), and performed RNA-Seq on ovarian mRNA. Our expectation was that divergence of *Hmr* between the orthologs might manifest as the failure of *sim-Hmr-FLAG* to complement the derepression of TEs in *Hmr^−^*.

As a control for the function of the transgenes, we compared the heterozygous wild type *Hmr^−^*/*Hmr^+^* to *Hmr^−^; ø{mel-Hmr-FLAG}/+*, as each genotype has one wild type copy of *Hmr^+^*. The majority of the upregulated TEs in *Hmr^−^* (Figure 3B) are suppressed by the *mel-Hmr-FLAG* transgene; however, 9 out of 182 families ranged from 2 to 9 times more highly expressed in *Hmr^−^; ø{mel-Hmr-FLAG}/+* than *Hmr^−^*/*Hmr^+^* (Figure 9B). This result suggests that *mel-Hmr-FLAG* does not fully complement the *Hmr* mutant phenotype, which may reflect its decreased expression compared to a wild type allele (Figure S2), though it is also possible that some differences may result from TE polymorphisms that remain between the strains. qRT-PCR also demonstrated that *sim-Hmr-FLAG* expresses in *D. melanogaster* at ∼3× the level of *mel-Hmr-FLAG* (Figure S2), a difference previously seen with *Lhr* transgenes [8]. Because *Hmr* is a negative regulator of TE expression, we suggest that this expression difference will not bias against our goal of identifying TEs that are not fully repressed by *sim-Hmr-FLAG*.

We did not find any difference in satellite DNA expression; however, we found 11 TE families that are differentially expressed between the transgenic genotypes (Figure 9C). Five are more highly expressed in *Hmr^−^; ø{mel-Hmr-FLAG}/+* with fold changes ranging from 2–3, of which 3 are incompletely repressed by *mel-Hmr-FLAG* in the control cross described above (*Transpac*, *Tirant*, and *Batumi*). The differential expression of these 5 families likely reflects the inability of *mel-Hmr-FLAG* to fully complement *Hmr^−^* and the higher expression level of *sim-Hmr-FLAG*.

More intriguing are 6 TE families that are 2–6× more highly expressed in *Hmr^−^; ø{sim-Hmr-FLAG}/+* than in *Hmr^−^; ø{mel-Hmr-FLAG}/+*, implying that *sim-Hmr-FLAG* is unable to fully complement the derepression of these elements. *BS* and *Doc6* (also known as *Juan*) elements are present at a mean frequency of about 0.1 in a population of Portuguese *D. melanogaster* [45] and have low pairwise identity in the reference genome [46], suggesting that they are likely active. The mean population frequencies of 4 of the other families (*BS3*, *Circe*, *Helena*, and *FW2*) are near 1, suggesting that these TEs are fixed and therefore currently inactive in *D. melanogaster*. *Helena*, though, appears to have been active more recently within *D. simulans* [*47*]. We suggest that *BS*, *Doc6* and *Helena* are candidates for future investigation of co-evolution with *Hmr* in either *D. melanogaster* or *D. simulans*.

### Transposable elements are upregulated in hybrids

**Figure 10:**
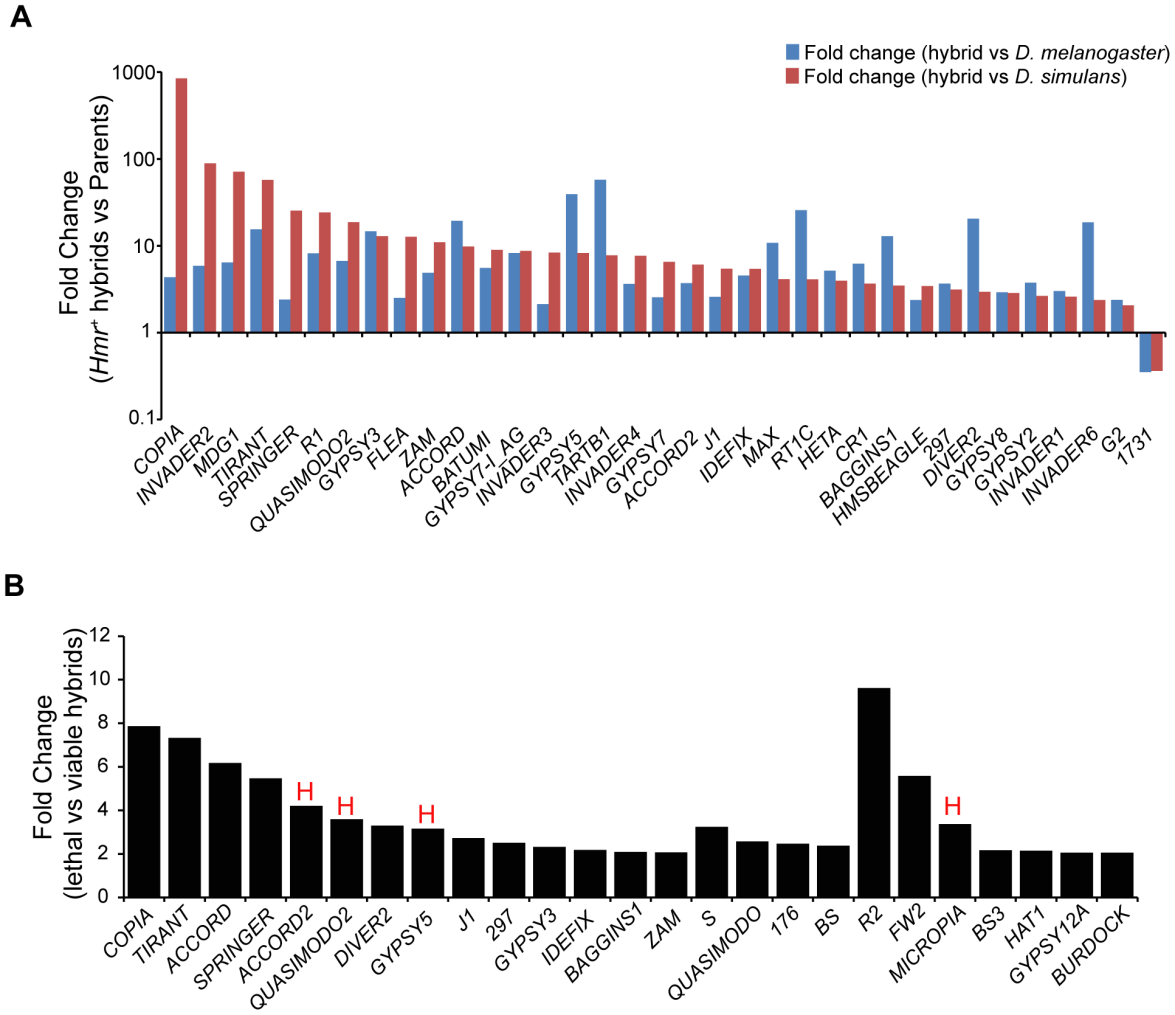
TE misregulation in hybrid males. (A) Fold change of TEs up- or downregulated ≥2-fold in *Hmr^+^* hybrid male larvae relative to both *D. melanogaster* and *D. simulans* male larvae. Uncharacterized *centroids* are not shown. (B) Fold change of TEs with significantly higher expression in lethal *Hmr^+^* versus viable *Hmr^−^* hybrid male larvae. “H” indicates TEs that are significantly upregulated in *Hmr^−^ D. melanogaster* male larvae compared to *Hmr^+^ D. melanogaster* male larvae from Figure 3D. Note the different Y axis scales between panels A and B. TE families include those resulting from mapping reads to the individual-insertion database, as well as families found only when reads were mapped to the consensus-sequence database. Reads unique to each TE class were mapped allowing for up to 3 mismatches.

In light of our discovery that *Lhr* and *Hmr* are required for TE repression within *D. melanogaster* and *D. simulans*, we investigated TE activity in lethal (*Hmr^+^*) hybrid male larvae. Because most TEs have different expression levels between *D. melanogaster* and *D. simulans*, we defined mis-regulated TEs as being at least two-fold higher than both parental species, as done in a previous analysis [48]. We found that 42 LTR and non-LTR elements are significantly upregulated in lethal (*Hmr^+^*) hybrid male larvae with 2 others being downregulated (Figure 10A; Table S15).

We next examined whether TE misregulation correlates with hybrid lethality by comparing the lethal *Hmr^+^* hybrid males to viable *Hmr^−^* hybrid males (Figure 10B, Table S16). The expression of 29 TEs is significantly lower in *Hmr^−^* hybrids. Because *Hmr* functions as a repressor of TEs in *D. melanogaster* male larvae (Figure 3C), these differences may reflect a general difference between lethal and viable hybrids rather than the presence or absence of *Hmr* activity. In fact, only 4 of the 29 TEs downregulated in *Hmr^−^* hybrid male larvae are upregulated in *Hmr^−^ D. melanogaster* male larvae (Table S4).

In addition, we found modest increases (2–4 fold) in the activity of 5 TE families in living hybrids. None of these are significantly upregulated in *Hmr^−^ D. melanogaster* male larvae (Table S4). They include *TAHRE* and may reflect higher levels of cell proliferation in viable hybrids. Taken together our results suggest that TE overexpression is unlikely to be causing hybrid lethality.

## Discussion

### Lhr and Hmr interact with HP1a

We and others previously reported that Lhr (also known as HP3) interacts with HP1a [6], [9], [12], [30]. Here we report that Hmr also interacts with Lhr, and both are present in a complex together with HP1a. Consistent with this interaction, many of the roles we report here for Lhr and Hmr have been described for HP1a, including localizing to heterochromatin, regulating TE and pericentric gene expression, and controlling telomere length [49]–[51]. However, unlike mutations in *Su(var)205* which enodes HP1a [52], mutations in *Hmr* and *Lhr* are viable. Furthermore, Hmr and Lhr do not localize to the 359 bp satellite which forms a substantial fraction of X-linked pericentric heterochromatin Figure 1; [ref. 8]. These findings suggest that Hmr and Lhr are not ubiquitous heterochromatin proteins, leaving open the intriguing question of what guides their localization specificity.

The interaction of Hmr and Lhr with HP1a has recently been independently reported [53; AA Alekseyenko and M. Kuroda, personal communication]. Thomae et al. [53] also report other findings similar to ours here including repressive effects of Hmr and Lhr on TEs in somatic tissues and their localization to telomeres. Several conclusions are similar between the two studies and with previously published conclusions. Thomae et al. [53] observe upregulation of TEs in hybrids but conclude that they are unlikely to be the direct cause of hybrid lethality, a conclusion we reach below using different methods. Their conclusion that hybrids are highly sensitive to *Hmr* dosage is in concordance with previous studies, such as the previous observation that a ∼9.7 kb *Hmr^+^* transgene causes dosage-dependent lethality to hybrid females [3]. This conclusion also fits well with the discovery that hybrids are highly sensitive to *Lhr* dosage [8].

One area of possible discrepancy is the viability effects and cellular phenotypes associated with *Hmr* and *Lhr* mutants versus RNAi knockdown. Thomae et al report a high rate of mitotic defects in *Lhr* RNAi knockdown tissue culture cells, yet we found that *Lhr^KO^* flies are almost fully viable (see Results), as are *Lhr* RNAi knockdown animals [53]. We also have not observed the lethality or morphological defects in *Hmr* mutants that are reported for *Hmr* RNAi cells and animals [53]. For example, Aruna et al. [4] found reduced longevity but no effect on viability up to eclosion of flies carrying the *Df(1)Hmr^−^* allele, a deletion of the 5′ end of *Hmr*. Further work is necessary to determine if these discrepancies reflect phenotypes associated with the use of RNA interference or differences between assaying whole animals versus tissue-culture cells, such as the aneuploid state of cultured cell lines [54].

### Rapidly evolving heterochromatin proteins and repetitive DNA variation

Several HIs involve heterochromatin proteins or heterochromatic sequences, leading to the suggestion that genetic conflicts between selfish DNAs and host fitness are an important force that is driving the evolution of HI [1], [2], [23], [55].

TE and satellite abundance varies widely among species and is a major contributor to genome-size variation. The evolutionary causes of this variation have been widely debated for many years [56]. When considering genetic conflict theories, it is important to first exclude alternative evolutionary causes of repetitive DNA variation. One explanation is neutrality, with repeat variation governed by mutational processes, in particular the balance between insertions and deletions [57]. Insertion/deletion models are particularly appropriate for inactive and degenerate TEs, and perhaps also for certain classes of satellites that are no longer homogenized by concerted evolution [58].

Selectionist models fit better for active repeats, and must be invoked if the adaptive evolution of heterochromatin proteins is proposed to reflect co-evolution with repetitive DNA. One model is that some repeats are co-opted for host functions. Drosophila's telomeric retrotransposons are a relevant example that is discussed below. We also consider three, non-mutually exclusive selective costs associated with repetitive DNA when discussing the evolution of *Hmr* and *Lhr*

One potential cost arises from the overall load of repetitive DNAs, including increased genome size and instability. A second is direct genetic conflict. We define genetic conflict here to refer to fitness costs imposed by selfish DNAs that have evolved specific mechanisms to increase their transmission [59]. Such conflicts could be caused by highly active individual repeats, for example during hybrid dysgenesis caused by introduction of a TE family into naive strains [60]. Finally, genetic conflicts can have indirect costs, such as pleiotropic fertility defects caused by repeat expansions involved in meiotic drive [61].

### *Hmr* and *Lhr* repress transposable elements

TEs define selfish DNA [56]. They infect most genomes, can self-mobilize and increase their copy number, and destabilize genomes via spontaneous mutations, ectopic recombination, and deleterious increases in genome size [62], [63]. Adaptive evolution of TE-defense genes can therefore be readily interpreted as the host species responding to the fitness cost of TEs [19].

Like *Hmr* and *Lhr*, many piRNA pathway genes are also evolving under positive selection [22]. This raises the possibility that *Lhr* and *Hmr* are co-evolving with the piRNA pathway proteins. However, the lack of major perturbations in the piRNA pool in *Lhr^KO^* suggests that Lhr and Hmr function downstream or independently of piRNA biogenesis. Piwi, guided by piRNA, has been proposed to recruit repressive heterochromatin components including HP1a and histone methyl transferases to transposable elements [51], [64]. One possibility is that Lhr and Hmr function downstream of HP1a to repress TEs via RNA degradation machinery such as the nuclear exosome [65].

We note that *Ago3* is moderately down-regulated in both *Lhr^KO^* (3.4 fold) and *Hmr^−^* (∼2 fold) (Tables S5, S6), likely because the gene is peri-centromeric. Two results demonstrate that this modest reduction in *Ago3* cannot explain the broad effects on TEs in *Hmr* and *Lhr* mutants. First, *Ago3* expression is unaffected in *D. simulans Lhr^1^*, which also shows widespread TE derepression. Second, *Ago3* mutants have major disturbances to their piRNA pool [38], which we did not observe in *Lhr^KO^* (Figure 7).

### *Hmr* and *Lhr* regulate telomeres

While TE repression is typically viewed in terms of genetic conflicts, the relationship between *Lhr*, *Hmr* and the telomeric TEs resembles symbiosis. These TEs have been domesticated by Drosophila species for tens of millions of years to serve a vital host function, and thus are not considered selfish DNA [33], [66]. The telomeric TEs were among the most strongly derepressed in *Hmr* and *Lhr* mutants, in some cases more than 100 fold. We also observed increases in *HeT-A* DNA copy number in *Hmr* and *Lhr* stocks. Increased telomeric TE expression does not necessarily increase *HeT-A* DNA copy number and cause longer telomeres, suggesting that multiple factors control telomere length [67]. If so, then *Lhr* and *Hmr* must control multiple processes at the telomere. This is supported by the localization of both proteins to the telomere cap, a protective structure that prevents telomere fusions [28]. The strong reduction in *Lhr^KO^* of piRNAs from three TAS-repeat containing sub-telomeric piRNA clusters is particularly intriguing. piRNA production from clusters is dependent on them maintaining a heterochromatic state [68], which could explain why *Lhr* is required for TAS piRNA expression while it acts as a repressor in most other circumstances.

### *Hmr* and *Lhr* regulate species-specific satellite DNAs

We discovered several striking examples that suggest species-specific co-evolution of *Hmr* and *Lhr* with satellite DNAs. We found that *D. melanogaster* Hmr and Lhr proteins localize to and repress transcripts from GA-rich satellites. GA-rich satellites are ∼8 fold less abundant in *D. simulans* [13] but are cytologically detectable; nevertheless we find that sim-Lhr does not localize to them. GA-rich satellites also have low abundance in the outgroup species *D. erecta* [13], implying that the differential abundance with *D. simulans* reflects an increase in *D. melanogaster*. Similarly we discovered that mel-Lhr-HA localizes to AACAC in *D. melanogaster*, a repeat that is absent in *D. simulans* [69]. Furthermore, we detected moderate up-regulation of several other satellite transcripts only in *D. melanogaster*. Our results suggest that *Lhr* and *Hmr* may have evolved in *D. melanogaster* to mitigate the deleterious consequences of satellite expansion, which can include ectopic recombination, increased genome size, and destabilized chromosome segregation [16], [70].

Satellite transcripts have been reported from various tissues in wild type *D. melanogaster* [71], [72] but little is known about their production. They could be products of either non-specific transcription or read-through from adjacent TEs. Increased levels of satellite transcripts are observed in *D. melanogaster spn-E* mutants, suggesting that RNA interference or piRNA pathways control satellite transcript levels [21].

### Is the adaptive evolution of *Hmr* and *Lhr* driven by diverging heterochromatic repeats?

We find that at a broad scale, *Lhr* and *Hmr* from both *D. melanogaster* and *D. simulans* regulate heterochromatic repetitive DNAs but very few genes. This finding is consistent with previous analyses demonstrating that some functions of these genes are conserved between species [4], [7]–[9]. But many of the repeats regulated by *Lhr* and *Hmr* are rapidly evolving, raising the question of whether specific repetitive DNAs are directly driving the adaptive evolution of the *Lhr* and *Hmr* coding sequences between species. A simple prediction is that *D. simulans* orthologs should fail to fully repress such repeats when placed into *D. melanogaster*, a prediction that we tested for *Hmr*.

The *BS* non-LTR retrotransposon is significantly derepressed in *D. melanogaster Hmr^−^* and *Lhr^KO^*, and in *D. simulans Lhr^1^* mutants. Interestingly, *BS* appears to be transpositionally active in *D. melanogaster* but inactive in *D. simulans* [73]. One interpretation is that *BS* was active in the common ancestor and regulated by *Hmr* and *Lhr*. The genes would continue to co-evolve with *BS* in *D. melanogaster*, making the *sim-Hmr* ortholog less effective at repressing *BS* elements in *D. melanogaster*. In this scenario *Hmr* and *Lhr* are engaged in a recurrent genetic conflict with *BS* elements that leads to their sequence divergence. Consistent with this prediction we found significantly higher expression in *Hmr^−^; ø{sim-Hmr-FLAG}/+* compared to *Hmr^−^; ø{mel-Hmr-FLAG}/+*.

*Copia* shows a different pattern, with ∼20-fold up-regulation in *Lhr^KO^* but only ∼2-fold in *Lhr^1^* (and only when mapping to the consensus-sequence database), as well as significant derepression in *Hmr^−^*. *Copia* expression level can be high in *D. melanogaster* but is variable among populations. In contrast, *copia* elements in *D. simulans* typically contain deletions in regulatory elements required for expression, and transcripts are undetectable by Northern blot analysis [74]. These results suggest that *Hmr* and *Lhr* could be *D. melanogaster* host factors that defend against a TE that is currently active within the species. However, *copia* was fully repressed in *Hmr^−^; ø{sim-Hmr-FLAG}/+*, demonstrating that adaptive divergence of *Hmr* by itself does not affect *copia* regulation.

Overall, we found surprisingly few cases of overexpression associated with *Hmr* divergence, including no effects on satellite DNAs (Figure 9). We also note that most of the TEs identified other than *BS* and *Doc6* are likely transpositionally inactive in *D. melanogaster* [45], which makes it more challenging to fit a scenario of direct and recurrent evolution between *Hmr* and specific TEs.

We suggest several possible interpretations of these results. One is that *Hmr* and *Lhr* adaptive divergence is in fact driven largely or solely by *BS* and/or *Doc6*, a hypothesis that will require understanding the mechanism by which *Hmr* and *Lhr* affect expression of these TEs. Second is that *Hmr* and *Lhr* may be co-evolving with other genes, and that multiple diverged genes need to be replaced simultaneously in order to detect their effects on other TEs and satellite DNAs. Third is that more sensitive assays are needed, for example monitoring TE transposition rates over multiple generations. A fourth possibility is an alternative to genetic conflict scenarios that arises from population-genetic models. These models suggest that the fitness costs of individual TE families are likely extremely weak under most circumstances. The adaptive evolution of repressor proteins may therefore reflect the cumulative load of repeats within a genome [22]. This alternative view could be applicable to *Hmr* and *Lhr* since they repress a large number of TEs and satellites. Finally, *Hmr* and *Lhr* may have additional unidentified phenotypes that are also the targets of adaptive evolution.

### Repeat load, adaptation and hybrid incompatibilities

*D. simulans* has a smaller genome with ∼4-fold less satellite DNA [13], [14] and significantly fewer TEs [24], [75] compared to *D. melanogaster*. This large difference in repeat content between *D. melanogaster* and *D. simulans* may have wider consequences. We found reduced expression from pericentric heterochromatin genes in *Hmr* and *Lhr* mutants in *D. melanogaster*. This reduction may reflect the fact that pericentric genes have evolved to use heterochromatin proteins such as Lhr and Hmr to maintain gene expression in a repeat-rich environment [76]. Pericentric genes in species with fewer repeats would presumably not require these proteins. Consistent with this model, we found that *Lhr* loss in *D. simulans* has a negligible impact on pericentric gene expression. This finding suggests that *Lhr* and *Hmr* have an adaptive role in blocking effects on gene expression arising from increasing repetitive DNA copy number.

If each genome is uniquely adapted to its repetitive DNA content, then the shock of hybridization may lead to misregulation of TEs and satellites. TEs are activated in various animal and plant hybrids but the consequences, if any, for hybrid fitness are largely unclear [77]. We found substantial TE misregulation in hybrid male larvae (Figure 10A). Since these hybrids are agametic [78], this TE expression comes from somatic tissues. The fitness cost of this upregulation is unclear as somatic TE overexpression is not necessarily lethal within *D. melanogaster* [79], [80]. Comparison of lethal *Hmr^+^* and viable *Hmr^−^* hybrid males demonstrates that lethal hybrids have more TE expression (Figure 10B) than the viable hybrids, which in turn have more TE expression than either of its parents. However, this TE misregulation seems unconnected with *Hmr* as the TEs differentially expressed between *Hmr^+^* and *Hmr^−^* hybrid male larvae are largely distinct from those between *Hmr^+^* and *Hmr^−^ D. melanogaster* male larvae. Further, while *Hmr^−^* causes rampant TE over-expression within *D. melanogaster*, it is associated with reduced TE levels in hybrids. These observations argue that the TE derepression in hybrids is unrelated to the pure species function of *Hmr*. This finding is consistent with previous genetic studies that demonstrate that the wild type *Hmr^+^* allele causes hybrid lethality and thus behaves as a gain-of-function allele in hybrids [81], [82]. More generally it underscores the unique nature of the hybrid genetic background [1]. Somatic TE overexpression may result from breakdown in the siRNA or piRNA pathways due to incompatibilities among multiple rapidly evolving TE regulators.

One clear example is known where a species-specific difference in a satellite DNA causes incompatibility between Drosophila species [83]. But the toll caused by heterochromatic differences may more commonly be indirect, as heterochromatin proteins diverge in response to changes in heterochromatic DNA repeats. Recent work suggests that hybrid female sterility may be caused by incompatibilities among rapidly evolving piRNA proteins rather than by species-specific differences in TEs [48]. We suggest that the role of *Hmr* and *Lhr* in regulating the activity of three highly dynamic classes of heterochromatin has led to their recurrent adaptive evolution, and secondarily, to their involvement in interspecific hybrid lethality.

## Materials and Methods

### Construction of the *Lhr^KO^* mutant

We used the pW25 donor vector and ends-out homologous recombination method to make an *Lhr* mutant allele [84]. The donor vector was designed to recombine a *w^+^* marker into *Lhr* and simultaneously remove 26 bp of the coding region. iProof (Biorad) was used to PCR amplify two genomic fragments from *y; cn bw sp* (*D. melanogaster*) genomic DNA. The 3768 bp *Lhr* upstream fragment, including 128 bp of the coding region of *Lhr*, was amplified with primers LUF-Fwd: 5′- ttggcgcgccAACAGGGTCGGCTGTCACATTT and LUF-Rev: 5′-ttggcgcgccGCGAGCATCTCCATGAGCAG (Tm = 63°C) and cloned into the *Asc*I site of pW25 using the underlined sequences. The 3935 bp *Lhr* downstream fragment that includes 806 bp of the *Lhr* coding region was amplified with primers LDF-Fwd: 5′-AAGCGGCCGCAGGTGGAGCCCAAAATGGACG and LDF-Rev: 5′- AAGCGGCCGCCACACATTGCGAATGCA G AAA (Tm = 65°C) and cloned into the *Not*I site using the underlined sequences. Restriction digestion was used to pick a clone in which the 2 inserts and the *mini-white* gene were in the same orientation.

The construct was injected into a strain of *w^1118^* (Genetic Services) and a transgenic line, *P{w^+^, Lhr-KO}5-1*, with a lethal insertion on the X chromosome was obtained. *P{w^+^, Lhr-KO}5-1/FM6* females were crossed to *y w; P{ry^+^, hs-flpase}*, *P{v^+^ hs-I-Sce}/TM6*, *Ubx* males. Two to three day-old larvae were heat shocked and *P{w^+^, Lhr-KO}5-1/y w P{ry^+^, hs-flpase}*, *P{v^+^ hs-I-Sce}*/+ female progeny were crossed to *w^1118^* males. Rare *w* ***^+^*** sons were screened for homologous recombination events by PCR. Primer pairs *Lhr-f1* 5′- TTCGCACGTTGTGTTCAAGTAA-3′, /Lhr-r1 5′-GTAGCTTTCTCTTGGCGCTCTT-3′ and Lhr-f2 5′- AACGTGCTCGTAGCTTTGGT-3′/, Lhr-r2 5′-TCGCGAAAATACTTCCGTCT-3′ (Tm = 58°C) produce no amplicons in the presence of the *white* insertion. Attempts to remove the *w^+^* marker by *Cre* recombination were unsuccessful and the *w^+^*-disrupted *Lhr* locus was designated as *Lhr^KO^*.

To test the genetic effects of this mutation, we took advantage of a recent observation that a deficiency chromosome which deletes *D. melanogaster Lhr* can weakly rescue *D. melanogaster-D. mauritiana* hybrid males to the pharate adult stage [8]. When we crossed *Lhr^KO^* homozygous females to *D. mauritiana* males at 18°, we obtained 10.6% rescue of live males (17 males and 161 females). The stronger rescue observed here may be due to the fact that the mothers of the cross were homozygous for the *Lhr^KO^* allele, since *Lhr* likely has strong maternal expression based on its high protein abundance in early embryos [8].

### *Hmr* transgenes

A *D. melanogaster Hmr-FLAG* transgene was made by inserting a 3× FLAG tag sequence [85] immediately upstream of the stop codon of *Hmr* using fusion PCR into plasmid p72, which is a pCaSpeR2 vector containing a ∼9.7 kb fragment of the *Hmr* region [3]. Two *Hmr* fragments (L-arm and R-arm) were amplified from p72 with iProof polymerase by using primer pairs 739/738 and 736/740, respectively. The primers 738 and 736 contain sequence encoding the FLAG tag and partially overlap to allow fusion in the subsequent stage. The primers 739 and 740 were combined with L-arm and R-arm products to produce a fused partial fragment of *Hmr* containing the 3× FLAG sequence. This fragment was cloned into the pCR-BluntII-Topo vector (Invitrogen) and sequenced completely between the *Avr*II and *Kpn*I restriction sites. The *Avr*II/*Kpn*I fragment was then cloned into the corresponding sites of the p72 plasmid. A 300 bp fragment containing the *attB* site was then PCR amplified from plasmid *pTA-attB* (gift from Dr. Michele Calos) using primers 502 and 503 and cloned into the *Not*I site. This fragment was digested with *Not*I (on the ends of 502 and 503), gel purified, and inserted into the *Not*I site of the plasmid containing *Hmr-FLAG*. We refer to this transgene as *mel-Hmr-FLAG*.

A *D. melanogaster Hmr-HA* transgene was made by inserting a 3XHA epitope tag between codons 466 and 467 of *Hmr*. Primers 215/1246 and 1247/495 were used to amplify 573 and 316 bp fragments, respectively. Primers 1246 and 1247 overlap and encode the HA tag. Fusion PCR containing these 2 products and primers 215/495 was performed. The PCR product was cloned into pCR-Blunt II-TOPO, and the insert was checked by sequencing. The insert was then cloned using *Spe*I and *BsiW*I back into a modified p72 containing an *attB* site inserted into the *Not*I site. The orientation and presence of the HA tag were checked by double digests and PCR. We refer to this transgene as *mel-Hmr-HA*.

A *D. simulans Hmr-FLAG* transgene was made by inserting the 3× FLAG tag sequence upstream of the stop codon in p89, a pBluescript II KS(+) plasmid containing the *D. simulans Hmr* insert that was used for the p92 transformation construct in [7]. Primers 751/753 and 750/752 were used to amplify 1.3 kb and 1.8 kb fragments of the insert, respectively, which were then joined by fusion PCR using primers 750/751. The fusion PCR product was cloned into pCR-Blunt II-TOPO and confirmed by sequencing. The insert was designed to have an *Hpa*I site near one end and a *Not*I site near the other. The *Not*I site was destroyed during cloning; however, the pCR-Blunt II-TOPO vector contains a *Not*I site within 40 bp of the destroyed sequence. The insert was then cloned back into p89 using *Hpa*I and *Not*I. The orientation of the insert, as well as the addition of the FLAG tag, was checked by double digest with *Cla*I and *Hpa*I. The *D. simulans Hmr-FLAG* insert was then removed as a *Sac*II fragment. Klenow enzyme was used to fill-in the ends to allow cloning into the *Stu*I site of pCaSpeR2 containing an *attB* site inserted at its *Not*I site. We refer to this transgene as *sim-Hmr-FLAG*.

The *D. simulans Hmr-HA* transgene was made from plasmid p89 by inserting the HA tag at the region orthologous to *mel-Hmr-HA* [7]. Primers 135/1365 and 1247/1364 were used to amplify 861 bp and 827 bp fragments, respectively, from the p89 template, and were fused together using primers 1364/135. The fusion PCR product was then cloned into pCR-Blunt II-TOPO and the entire insert was checked by sequencing. The insert was then cloned back into p89 using *Spe*I and *Blp*I. Blunt end ligation, used for *sim-Hmr-FLAG* above, proved inefficient for transferring the insert into the transformation vector. Therefore an *Xba*I site was added to the 3′ end of *Hmr-*HA by amplifying the entire insert using primers 1402/1403. The PCR product was then gel purified and cloned back into pCR-Blunt II-TOPO. The polylinker contains an *Xba*I site 5′ to the insert, allowing us to clone the entire insert into the *Xba*I site of pCaSpeR2 containing an *attB* site inserted at its *Not*I site. We refer to this transgene as *sim-Hmr-HA*.

Oligonucleotides for *Hmr* transgenes (all written 5′-3′). 739: AGCCAAATTGCCGACAGTAGCCAAG; 738: ATCGATGTCATGATCTTTATAATCACCGTCATGGTCTTTGTAGTCAGGCGGTGGCGGATTGACCTTG; 736: GACGGTGATTATAAAGATCATGACATCGATTACAAGGATGACGATGACAAGTAGCTCTCGAAACTTTTGGCACACGTAG; 740: TTGTACTGCCATTAGGTATAGCTAACCATCC; 502: AAACCCGCGGCCGCATGCCCGCCGTGACCGTC; 503: AAACCCGCGGCCGCGATGTAGGTCACGGTCTCG; 152: TCTTCTTAGACTGCGGGTTG; 215: CAGCGCATGCGCGGCACCGTAT; 1246: ATAGTCCGGGACGTCATAGGGATAGCCCGCATAGTCAGGAACATCGTATGGGTACATTGCACTGTTGGTCATGCTCGT; 1247: TCCCTATGACGTCCCGGACTATGCAGGATCCTATCCATATGACGTTCCAGATTAC;GCTAGCACTGCCACAAGCATTGG; 495: GACACGCCCGTTCCCATAGT; 751: ACAGCGATTTGCGCAAGCCG; 753: TCGATGTCATGATCTTTATAATCACCGTCATGGTCTTTGTAGTCAGGCGGTGGCGGATTTGCCTTCTTGGCGTATTTAGA; 750: GTGAATTGTAATACGACTCACTATAGGGCG; 752: GACGGTGATTATAAAGATCATGACATCGATTACAAGGATGACGATGACAAGTAGCTCTCGAATCATTGGCACACG; 135: GAGGAGGACCCCACCTATAACTAC; 1365: ATAGTCCGGGACGTCATAGGGATAGCCCGCATAGTCAGGAACATCGTATGGGTATGCACTGTTAGAAATGCTTGTGCTG; 1364: GCTGGCAATTTGGACTTTGT; 1402: GCGGGCGGTCATTATTAA; 1403: TATCTAGAGCGGCCGCGAGCTCTAATA.

### Transgenic fly lines

φC31-mediated transgenesis was performed by Genetic Services using the *P{CaryP}attP2* integration site at cytological position 68A4 [86]. Site specificity of integration was checked by PCR assays described in references [8], [87]. *D. melanogaster* transformants were recognized by their w^+^-eye color and were crossed to a *y w* strain. Wild type activity of the *Hmr-HA* and *Hmr-FLAG* transgenes was tested for complementation of an *Hmr* rescue mutation in hybrids as done previously for *Hmr^+^* transgenes [3], [7]. Here we crossed *Df(1)Hmr^−^*, *y w v/FM6; ø{mel-Hmr-HA}/+* females to *D. simulans w^501^* males. We recovered 193 *w^501^/Y; +/+* hybrid males but only 1 *w^501^/Y; ø{mel-Hmr-HA}/+* hybrid male, demonstrating that the transgene is *Hmr^+^*. Likewise, we crossed *Df(1)Hmr^−^*, *y w v; ø{mel-Hmr-FLAG}/+* females to *D. simulans v* males, and recovered 451 *v* females, 258 *w* males and only 3 *w^+^* males.

### Drosophila strains

*Lhr^KO^* was outcrossed to *w^1118^* for six generations. Sibling crosses were then used to generate a homozygous *w^1118^*; *Lhr^KO^/Lhr^KO^* (abbreviated as *Lhr^KO^*), a heterozygous *Lhr^KO^/+*, and a wildtype *w^1118^*; *Lhr^+^/Lhr^+^* line (abbreviated as *Lhr^+^*). All experiments with *Lhr* in this paper use these matched mutant and sibling controls unless otherwise specified. The *D. simulans Lhr^1^* allele is caused by an insertion in the 5′ UTR and appears to make no transcript by RT-PCR [6]. *Lhr^1^* was outcrossed to the inbred wild-type line *w^501^* for 3 generations to generate the stock *w^501^; Lhr^1^* (abbreviated as *Lhr^1^*) and *w^501^*, *Lhr^+^* (abbreviated as *Lhr^+^*). *Lhr-HA* transgenes were described previously [8]. *y w* F10 was created by single-pair matings between siblings for 10 generations.

We refer to the *P{EPgy2}Hmr^3^* allele that is marked with *y^+^* and *w^+^* described in [4] as *Hmr^3^*. *Df(1)Hmr^−^*, *y w v*, abbreviated as *Df(1)Hmr^−^*, is described in [88]. In order to match backgrounds for the *Hmr* RNA-Seq experiments, the *Hmr^3^* stock and the transgenic lines (*mel-Hmr-FLAG* and *sim-Hmr-FLAG*) were outcrossed to *y w* F10 for 6 generations and then made homozygous.

### Fertility assays

Individual 1–2 day old virgin *Lhr^KO^* and *Lhr^KO^/+* sibling females, obtained from crosses of *Lhr^KO^/+* at 27°C, were crossed to two *w^1118^* males. Flies were transferred to a fresh vial every 5 days for 15 days. Vials in which either the female or both males were missing or dead were not scored or transferred. To create the heteroallelic siblings *Lhr^KO^/Df(2R)BSC44* and *Lhr^KO^/SM6a*, *Lhr^KO^/Lhr^KO^* were crossed to the *Lhr^−^* deletion stock *Df(2R)BSC44/SM6a* [6]. The fertility assay was carried out as above except vials were flipped every 4–5 days.

### Hatch rate assays

*Lhr^KO^/+* or *Lhr^KO^/Lhr^KO^* females were crossed to *w^1118^* males at 27°C. Egg lays were carried out on grape juice/agar plates for 3 hour periods at either 2–3 days, 5–6 days or 10–11 days after eclosion of the female parents. The plates were maintained at 27°C and monitored over the next 24–36 hours for hatched eggs.

### Crosses for generating *Hmr* genotypes for RNA-Seq of ovarian mRNA

*y w Hmr^3^; +/+* females were crossed to *y w; ø{mel-Hmr-FLAG}/ø{mel-Hmr-FLAG}* males. F1 males were crossed to *Df(1)Hmr^−^/FM6*; *+/+* females to generate both *y w Hmr^3^/Df(1)Hmr^−^*; *ø{mel-Hmr-FLAG}/+* and *y w Hmr^3^/Df(1)Hmr^−^*; *+/+*. Similarly, *y w Hmr^3^; +/+* females were crossed to *y w*; *ø{sim-Hmr-FLAG}/ø{sim-Hmr-FLAG}* males. F1 males were crossed to *Df(1)Hmr^−^/FM6*; *+/+* females to generate *y w Hmr^3^/Df(1)Hmr^−^*; *ø{sim-Hmr-FLAG}/+*. Lastly, *y w*; *+/+* females were crossed to *y w*; *ø{mel-Hmr-FLAG}/ø{mel-Hmr-FLAG}* males. F1 males were crossed to *Df(1)Hmr^−^/FM6*; *+/+* females to generate the heterozygous wildtype control, *y w*/*Df(1)Hmr^−^*; *+/+*. These crosses were done at 27°C and in triplicate to generate 3 biological replicates.

### Crosses for generating pure-species and hybrid samples for RNA-Seq of larvae

The *Df(1)Hmr^−^*, *y w v/FM7i*, *P{w+mC = ActGFP}JMR* stock (abbreviated as *Df(1)Hmr^−^/FM7i*, *GFP*) was described previously [88]. A stock with the matching *Hmr^+^* genotype, *y w v*/*FM7i*, *P{w+mC = ActGFP}JMR* (abbreviated as *Hmr^+^/FM7i*, *GFP*) was created by crossing *y w v/Y* males with *Df(1)Hmr^−^/FM7i*, *GFP* females. *FM7i*, *GFP/Y* males from this *Hmr^+^* stock were then crossed to *Df(1)Hmr^−^/FM7i*, *GFP* females for 10 generations in order to make the autosomal backgrounds comparable between the two stocks.

To generate hybrids, *Df(1)Hmr^−^/FM7i*, *GFP* or *Hmr^+^/FM7i*, *GFP* were crossed to *v/Y D. simulans* males. For each cross, 6 replicates were made each containing 25 0–12 hour-old virgin females and 50 4–6 day-old virgin males. Hybrid larval sons not carrying the balancer were selected by their *y^−^* mouth hook and *GFP^−^* body phenotypes. Additionally, some crosses were allowed to develop to ensure that only *Df(1)Hmr^−^* crosses produced hybrid sons. To generate *D. melanogaster* samples, 3 replicates of 10 *Df(1)Hmr^−^/FM7i*, *GFP* or *Hmr^+^/FM7i*, *GFP* virgin females were crossed to 15 *FM7i*, *GFP/Y* males. Larval sons not carrying the balancer were selected by *y^−^* and *GFP^−^* phenotypes. To generate *D. simulans* samples, 3 replicates of 10 *y w D. simulans* virgin females were crossed to 15 *v/Y D. simulans* males. Larval sons were selected by *y^−^*.

### Preparation of protein lysates for semi-quantitative western blots

50 mg of 1–17 hr embryo collections were dounced 30 times with a tight pestle in 500 ul buffer A1 (15 mM HEPES, pH = 7.5; 15 mM NaCl; 60 mM 1M KCl; 4 mM MgCl_2_; 0.5% TritonX-100; 0.5 mM DDT) and then centrifuged for 5 minutes at 4°C. The pellet was washed with 500 µl buffer A1 and centrifuged. This process was repeated another two times. The pellet was lysed by douncing in 200 µl SDS lysis buffer (500 µl 10% SDS, 200 µl 1M Tris, pH = 8.0, 40 µl 0.5M EDTA, 100 µl 100× protease inhibitor, 10 µl 0.5M EGTA, 50 µl 100 mM PMSF, 9.1 ml water). The lysate was allowed to rotate at 4°C for 20 minutes and then centrifuged. The supernatant was removed, quantitated using the Bradford assay and was run on an SDS-PAGE gel.

### Anti-Lhr antibodies and western blots

An Lhr cDNA was cloned into pDEST17 (Invitrogen). The expressed protein from *E. coli* was purified using Ni-Ag beads under denaturing conditions (8M urea), dialyzed down to 2M urea and injected into rabbits (Cocalico). The antisera was then purified by coupling purified His-Lhr to CnBr-activated Sepharose beads in the presence of 1% Triton-X and removing urea by dialysis. Antisera was eluted in 0.2 M glycine, pH 2.8 and then neutralized with 1M Tris, pH 8.5. The antibody failed to detect Lhr in immunofluorescent experiments but was used for Western blots in Figure S3 at 1∶4000 in 5% milk-TBST and HRP conjugated anti-rabbit secondary antibody at 1∶2000 dilution. HA-tagged Lhr was detected with 1∶1000 dilution of rat anti-HA (Roche, 3F10) and HP1a was detected with a 1∶700 dilution of mouse monoclonal supernatant (C1A9, DSHB).

### Co-immunoprecipitation

0∼16 hour-old embryos were collected, dechorionated and snap frozen in liquid nitrogen. Embryos were then resuspended to 10× embryo volume of Buffer A (10 mM Tris-Cl pH 8.0, 300 mM sucrose, 3 mM CaCl_2_, 2 mM Mg acetate_2_, 0.1% Triton X-100, 0.5 mM DTT, 0.5 mM PMSF) and homogenized with a dounce homogenizer. The homogenized lysate was centrifuged at 700 g for 10 minutes at 4° to pellet the nuclei. The supernatant was removed, the pelleted washed once in Buffer A, the nuclei centrifuged again and then resuspended in 1× embryo volume of Buffer MN (15 mM Tris-Cl pH 7.4, 250 mM sucrose, 60 mM KCl, 1.0 mM CaCl_2_, 0.5 mM DTT, 1× protease inhibitor cocktail). The nuclear lysate was sonicated briefly, micrococcal nuclease added to a concentration of 500 units/ml, and the chromatin digested for 1 hour at 4° with gentle agitation. EDTA and Triton X-100 were then added to a concentration of 5 mM and 0.1% respectively, to inactivate nuclease activity and solubilize the proteins, followed by incubation at 4° for 1 hour. After a second brief sonication, the digest was centrifuged at 12,000 g for 10 min at 4° and the supernatant was collected. 50 µl of the chromatin digest was diluted in IP Wash Buffer (50 mM Tris-Cl pH 7.4, 100 mM NaCl, 0.1% Triton X-100) with 1× protease inhibitor cocktail to a final volume of 125 µl per co-immunoprecipitation mixture. 15 µl of protein G-conjugated magnetic beads and 2–5 µl of antibody were added followed by incubation for 4 hours at 4° with gentle agitation. The beads were washed 3 times in IP Wash Buffer. The immunoprecipitated proteins were then eluted by boiling the beads in 1× Laemmli sample buffer for 5 minutes and analyzed by immunoblotting.

### RT-PCR and qRT-PCR assays

RNA extraction, cDNA synthesis and qRT-PCR assays were performed as in reference [8], using 2–5 µg of RNA. qRT-PCR experiments included three technical replicates of three separate biological replicates. Primers included: Lhr-f1 5′caccATGAGTACCGACAGCGCCGAGGAA, Lhr-r1 5′ ACACTTGGTTTTCGGCACATC CGC, Lhr-f2 5′ GTAGCTTTCTCTTGGCGCTCTT, Lhr-r2 5′ GTAAGTGAACTGAAGCTGC GTTGG, EDTP-F 5′GCTGGCAGGTGG TTACCGACA, EDTP-R 5′CGTGGCCAGGTTCA TGGATGA, Bap55-F 5′ CCGAGAGTC TCTTTGACAATGCA, and Bap55-R 5′GCCTCTT CGTACTCCTGCGA. Hmr-f1 5′ TAAGTTCGCCTTCCGCACATACC and Hmr-r1 5′ GACCAGAAACCTGAGTTGCTCCA. *HeT-A* and *RpL32* (also known as *Rp49*) transcript levels were measured with primers from reference [89].

### qPCR of *HeT-A* DNA copy number

The Invitrogen DNEasy kit was used to make genomic DNA from *Lhr^KO^* and *Lhr^+^* female carcasses that were free of ovarian tissue. Primers Het-s2 and Het-as2 amplify from the coding sequence of *HeT-A* [90]. *HeT-A* copy number was normalized to *RpL32* (also known as *Rp49*) copy number using primers from reference [89].

### RNA-Seq samples

For samples from ovaries, flies were kept at 27°C for several generations prior to and during the experiment. Freshly eclosed females were collected and aged 2–3 days and then transferred to fresh food with yeast paste for another 2–3 days. RNA was extracted, from ovaries dissected in chilled 1× PBS, using Trizol. Ovarian mRNA-Seq libraries were constructed at the Epigenomics Core Facility at Weill Cornell Medical College using the poly(A) enrichment method. Libraries were sequenced using the Illumina HiSeq2000 platform to produce 50 bp single reads which were then trimmed for quality and filtered to remove rRNA reads. One biological sample each from *Lhr^KO^* and *Lhr^+^* was duplexed and run in a single lane. 51,193,832 filtered reads were obtained for *Lhr^+^* and 41,688,028 reads for *Lhr^KO^*. Three biological replicates each of *D. simulans w^501^* and *Lhr^1^* ovarian mRNA libraries were run on a single lane and the number of filtered reads ranged from 36,472,726 to 43,449,879. For experiments with *Hmr*, two biological replicates were included for each genotype and all 8 samples were multiplexed in a single lane. The number of filtered reads for each sample ranged from 23,863,381 to 27,490,644. For larval samples, around 30 larvae were collected for each genotype and flash frozen in liquid N_2_. RNA was extracted from 2 biological replicates of each genotype using Trizol. Larval RNAseq libraries were generated and bar-coded using the TruSeq kit, and run in one lane of an Illumina HiSeq 2000 100 bp yielding 13,707,247 to 20,373,267 filtered reads per sample, except for one library which produced only 7,840,004 reads.

### RNA-Seq analysis

Reads mapping to either rRNA or repetitive DNA were filtered out using Bowtie [91] and the filtered reads were mapped to the unmasked *D. melanogaster* genome using Tophat [92]. The BAM file outputs were used by Cuffdiff with the -b option [93]. All *.fasta and *.gtf files were based on the release 5.68 of the *D. melanogaster* genome from ENSEMBL. To find differentially expressed genes in *D. simulans*, we aligned reads to the *D. melanogaster* genome with Tophat, allowing two mis-matches. While this approach could potentially reduce mapping ability for diverged genes, it allowed us to take advantage of the better assembly and annotation of the *D. melanogaster* genome.

To maximize the TEs considered in our analyses, we mapped reads to two different databases using Bowtie. First, reads were uniquely mapped to a database consisting of all the annotated TE insertions in the *D. melanogaster* and *D. simulans* genomes [48]; we refer to this as the individual-insertion database. While this database likely represents most TE families present in our stocks, some TEs may either be absent from the assembled genome or be represented by copies that are sufficiently diverged such that they impact our ability to correctly assess transcript levels. These elements include the telomeric element *TAHRE*, which has only a few insertions in the genome and is known to be absent from the reference genome since only two telomeres are included in the assembly [94]. Therefore we also mapped reads, allowing for either 0 mismatches when aligning reads from *D. melanogaster* or 3 mismatches when aligning reads from *D. simulans* or hybrids, to a database consisting of the consensus sequences of the annotated TEs and repeats found in Repbase as well as *de novo* predicted TEs generated by piler-DF using the 12 *Drosophila* genomes [48]; we refer to this as the consensus-sequence database. Only reads that mapped uniquely within the same family were included in the subsequent analyses of differential expression. Mismatches allowed for each alignment are mentioned in figure legends. Statistical significance of differential expression among TEs was calculated with F.E.T. in the DEG-seq package [95].

To analyze reads mapping to satellite DNAs, we built a database using a curated file from the Berkeley Drosophila Genome Project (http://www.fruitfly.org/sequence/sequence_db/na_re.dros) which itself was constructed from GenBank sequences. This file includes some mis-annotated TEs and non-satellite sequences. We counted reads that mapped to these repeats without any mismatches and calculated statistical significance of differential expression among satellites with F.E.T. in the DEG-seq package.

### Small RNA sequencing and analysis

Libraries were prepared as described but no oxidation was carried out [38]. Briefly, total RNA was extracted from 5–6 day old *Lhr^KO^* and *Lhr^+^* ovaries using the mirVANA kit (Invitrogen). Total RNA was size fractionated on a 15% Urea-PAGE gel to enrich for 18–29 nt small RNA, excised and eluted and then subjected to 2S rRNA depletion. This small RNA was ligated to a 3′ RNA adapter, gel purified, and then ligated to a 5′ DNA adapter. The adapter-ligated small RNAs were reverse transcribed and PCR amplified. The amplified PCR products were gel purified, quantified and sequenced in two lanes of a HISeq 2000 machine.

Only reads with a 3′ adapter were kept, which was then removed using a custom script [48]. These reads were binned by size as either miRNA/siRNA (17–22 nt) or piRNA (23–30 nt). rRNA, tRNA and snoRNA sequences were filtered from these reads and the remaining reads were further filtered to keep only those reads that mapped to either the unmasked genome,or the satellite DNA database described above, or Repbase consensus sequences [96]. These filtered reads included 89,953,149 piRNA reads and 40,859,119 siRNA reads in *Lhr^KO^*, and 120,143,855 piRNA reads and 36,388,192 siRNA reads in *Lhr^+^*.

piRNA reads were mapped uniquely to all *D. melanogaster* sequences from Repbase using Bowtie, allowing for one mismatch. Ping-Pong scores were calculated using reads mapped with up to 1 mismatch, as described in reference [48]. For mapping to piRNA clusters, we built an index using sequences extracted from the Release 5 DM3 genome on the UCSC genome database and GenBank with coordinates of individual piRNA clusters obtained from reference [41]. piRNA reads were uniquely mapped to piRNA clusters with zero mismatches and significance for differential expression was calculated using F.E.T implemented in DEG-seq. siRNA reads were mapped uniquely to all *D. melanogaster* sequences from Repbase with Bowtie, without allowing for any mismatches.

### Immuno-fluorescence and Immuno-FISH

Immunofluorescence and FISH were performed on embryos and ovaries as described in references [4], [83]. Polytene chromosomes were dissected in 0.7% NaCl, squashed, and fixed in 1.8% PFA, 45% acetic acid for 17 minutes. They were then washed in 1% Triton X in PBS for 10 minutes, then washed in 5% milk in PBS for 1 hour, incubated with primary antibody overnight at 4°C, washed in 5% milk in PBS for 10 minutes, incubated with secondary antibody for 1 hour at room temperature, and then washed for 10 minutes in buffer A (0.15M NaCl, 0.2% NP40 substitute, 0.2%Tween 20) followed by 10 minutes in buffer B (0.20M NaCl, 0.2% NP40 substitute, 0.2%Tween 20).

Rat anti-HA antibody (Roche, 3F10) was used at 1∶100, rat anti-Vasa (DSHB) was used at 1∶25, Fibrillarin (Abcam, Ab5281) was used at 1∶100, anti-HP1a antibody (C1A9, DSHB) was used at 1∶100. Alexa fluorophore-conjugated secondary antibodies were used to detect the primary antibody. Fluorescently labeled probes against GA-rich satellites, AACAC, 2L3L, 359 bp and dodeca were obtained from Sigma with sequences described in references [8], [83], [97]. Imaging was carried out using a Zeiss 710 confocal microscope at Cornell University's Microscopy and Imaging Facility.

### Yeast two-hybrid assays

A full-length coding-sequence plasmid of *D. melanogaster Hmr* was made by correcting 3 frame-shift errors in the RE54143 cDNA [3]. Two errors in exon 5 were replaced by ligating in a ∼1.6 kb *Xba*I-*Hind*III fragment from the LD22117 cDNA, followed by replacement of a 2172 bp *Nde*I-*Zra*I fragment from the p83 genomic clone [3]. The coding sequence was then PCRd out and cloned into pENTR/D-TOPO. The *D. simulans Hmr* CDS was PCRd out of cDNA and cloned into pENTR/D-TOPO. The *Lhr* plasmids and yeast two-hybrid destination vectors and assays are described in reference [6].

### Data access

Illumina sequence data from this study are available from the NCBI website under BioProject number PRJNA236022.

## Supporting Information

Figure S1 Lhr and Hmr colocalize with specific satellite sequences in ovaries. Nurse cell nuclei (blue) are stained with DAPI in all panels. Scale bars represent 5 µm. (A) mel-Lhr-HA (green) colocalizes with GAGAA(red, top panel) and AACAC (red, bottom panel) in the nurse cells of *Lhr^KO^/+*; *LhrHA/+* ovaries. Arrows point to overlaps between bright FISH and HA-staining foci. (B) mel-Hmr-HA (green) colocalizes with GAGAA (red) and (C) dodeca (red) in nurse cells of *Hmr^3^*; *mel-Hmr-HA/mel-Hmr-HA* ovaries in a subset of nuclei. Arrows point to overlaps between FISH signals and the brightly staining foci of mel-Hmr-HA. Two different egg chambers are shown for both dodeca and GAGAA.
(TIF)

Figure S2 qRT-PCR analysis of *Hmr-FLAG* transgenes. *Hmr* transcript levels in transgenic lines were compared to the host strain (*Hmr^−^*) and also to *Hmr^+/^* ^−^. The transgenes are heterozygous, therefore both the transgenic lines and *Hmr^+/−^* carry one copy of *Hmr^+^*. RNA was isolated from ovaries and *Hmr* expression levels were normalized relative to *RpL32*. Error bars represent standard error within 3 biological replicates. The difference in the expression level of *mel-Hmr-FLAG* and *sim-Hmr-FLAG* is significant (*p* = 0.009, two-tailed *t*-test with equal variance). Additionally, the expression of *mel-Hmr-FLAG* is significantly different than an endogenous copy of *Hmr* (*p* = 0.007, two-tailed *t*-test with equal variance).
(TIF)

Figure S3 The *D. melanogaster Lhr^KO^* allele generated by homologous recombination. (A) *Lhr* and flanking genes are shown, the red triangle labeled *w^+^* indicates the site of the insertion in the *Lhr^KO^* allele, which is predicted to be ∼4.7 kb based on the structure of the targeting vector. Products used in RT-PCR reactions in (B) are shown below the genes. *EDTP* gene is partial; *w+* insertion not to scale. (B) RT-PCR from adult females shows no *Lhr* transcript spanning the *w^+^* insertion (*Lhr-5′-w-3′*) in *Lhr^KO^*. A highly reduced amount of *Lhr* transcript is detected 3′ to the *w+* insertion (*Lhr-w-3′*). The flanking genes *Bap55* and *EDTP* are not affected. *w^1118^* was used as a *Lhr^+^* control. +, − indicates presence or absence of reverse transcriptase (RT). (C) Western analysis shows that *Lhr^KO^* produces no protein. A non-specific band indicated by the asterisk is used a loading control.
(TIF)

Figure S4 qRT-PCR analysis shows elevated *HeT-A* levels in *Lhr* mutants. qPCR was used to estimate the transcript levels of *HeT-A* relative to the gene *RpL32* in poly-A primed cDNA samples obtained from ovarian RNA from two different *Lhr^−^* backgrounds and matching controls. (A) Ratio of *HeT-A*/*RpL32* in *Lhr^KO^* vs. *Lhr^+^*, showing mean from 3 biological replicates. Significance of fold change was calculated using Welch's one-tailed *t*-test; *p*<0.05. (B) Ratio of *HeT-A*/*RpL32* in *Lhr^KO^/Df(2R)BSC44* vs. *Lhr^+^/Df(2R)BSC44*, showing mean from 4 biological replicates. Significance of fold change was calculated using the one tailed Wilcoxon rank sum test; *p*<0.05.
(TIF)

Figure S5 Localization of Hmr-HA and Lhr-HA to the telomeres is independent of dosage of endogenous copies. mel-Hmr-HA (green) in *Hmr^3^*; *Hmr-HA* (A–C) and mel-Lhr-HA (green) in *Lhr^KO/^+*; *Lhr-HA/+* (D) colocalize with HP1A (red) at the telomere cap on polytene chromosomes. mel-Hmr-HA shows a range of distributions at the telomere, including punctate (B) and continuous across the chromosome terminus (C). Scale bar is 1 µm.
(TIF)

Table S1 Eggs laid by *Lhr^KO^* mothers have a reduced hatch rate. Hatching of eggs laid by *Lhr^KO^/+* or homozygous *Lhr^KO^* mothers crossed to wild-type fathers was followed for 36 hrs after egg lay. For *Lhr^KO^/+*, 34 eggs from days 2–3, 289 from days 5–6 and 668 eggs from days 10–11 were counted. For *Lhr^KO^*, 46 eggs from days 2–3, 209 from days 5–6 and 287 eggs from days 10–11 were counted. The significance of the difference in the hatch rates of the eggs laid by *Lhr^KO^* and *Lhr^KO^/+* mothers was calculated by one tailed F.E.T., and was significant at all time points (*p*<10^−4^).
(DOCX)

Table S2 DEG-seq output measuring the statistical significance of the differences in TE expression between *Lhr^KO^* and *Lhr^+^* ovaries based on reads uniquely mapped with no mismatches to either the individual-insertion or consensus-sequence TE databases.
(XLSX)

Table S3 DEG-seq output measuring the statistical significance of the differences in TE expression between *Hmr^−^* and *Hmr^−^/Hmr^+^* ovaries based on reads uniquely mapped with no mismatches to either the individual-insertion or consensus-sequence TE databases.
(XLSX)

Table S4 DEG-seq output measuring the statistical significance of the differences in TE expression between *Hmr^+^* and *Hmr^−^* male larvae based on reads uniquely mapped with up to three mismatches to either the individual-insertion or consensus-sequence TE databases.
(XLSX)

Table S5 Cuffdiff output measuring the statistical significance of the differences in protein-coding gene expression between *Lhr^KO^* and *Lhr^+^* ovaries, based on reads uniquely mapped with up to 2 mismatches.
(XLSX)

Table S6 Cuffdiff output measuring the statistical significance of the differences in protein-coding gene expression between *Hmr^−^* and *Hmr^−^/Hmr^+^* ovaries, based on reads uniquely mapped with up to 2 mismatches.
(XLSX)

Table S7 DEG-seq output measuring the statistical significance of the differences in satellite DNA expression between *Lhr^KO^* and *Lhr^+^* ovaries, based on reads uniquely mapped with no mismatches.
(XLSX)

Table S8 DEG-seq output measuring the statistical significance of the differences in satellite DNA expression between *Hmr^−^* and *Hmr^−^/Hmr^+^* ovaries, based on reads uniquely mapped with no mismatches.
(XLSX)

Table S9 DEG-seq output measuring the statistical significance of the differences in piRNAs uniquely mapping with up to one mismatch to TE consensus sequences from Repbase between *Lhr^KO^* and *Lhr^+^* ovaries.
(XLSX)

Table S10 Ping-pong scores in *Lhr^KO^* and *Lhr^+^* ovaries calculated as described in [48]. Those with fold-change difference >2 are indicated in bold.
(XLSX)

Table S11 DEG-seq output measuring the statistical significance of the differences in piRNAs uniquely mapping with up to one mismatch to piRNA clusters between *Lhr^KO^* and *Lhr^+^* ovaries.
(XLSX)

Table S12 DEG-seq output measuring the statistical significance of the differences in siRNAs uniquely mapping with no mismatches to TE consensus from Repbase between *Lhr^KO^* and *Lhr^+^* ovaries.
(XLSX)

Table S13 Cuffdiff output measuring the statistical significance of the differences in protein-coding gene expression between *D. simulans w^501^* and *Lhr^1^* ovaries, based on reads uniquely mapped to the *D. melanogaster* genome with up to 2 mismatches.
(XLSX)

Table S14 DEG-seq output measuring the statistical significance of the differences in TE expression between *D. simulans w^501^* and *Lhr^1^* ovaries based on reads uniquely mapped to either the individual-insertion or consensus-sequence TE databases, while allowing for no mismatches for insertions and up to three mismatches for consensus.
(XLSX)

Table S15 DEG-seq output measuring the statistical significance of the differences in TE expression of male larvae between *D. melanogaster* and *D. melanogaster- D. simulans* hybrids (Sheets A and B), and between *D. simulans* and *D. melanogaster-D. simulans* hybrids (Sheets C and D). Unique reads were mapped to the individual-insertion (Sheets A and C) and consensus-sequence (Sheets B and D) TE databases, allowing for up to 3 mismatches.
(XLSX)

Table S16 DEG-seq output measuring the statistical significance of the differences in TE expression between viable *Hmr^−^/Y* hybrids and lethal *Hmr^+/^Y* dying hybrids. Unique reads were mapped to the individual-insertion and consensus-sequence TE databases, allowing for up to 3 mismatches.
(XLSX)
