# Supplementary figures and images for: The Hmr and Lhr Hybrid Incompatibility Genes Suppress a Broad Range of Heterochromatic Repeats
Source: PLoS Genet. 2014 Mar 20;10(3):e1004240. doi: 10.1371/journal.pgen.1004240 (PMC3961192; doi:10.1371/journal.pgen.1004240)

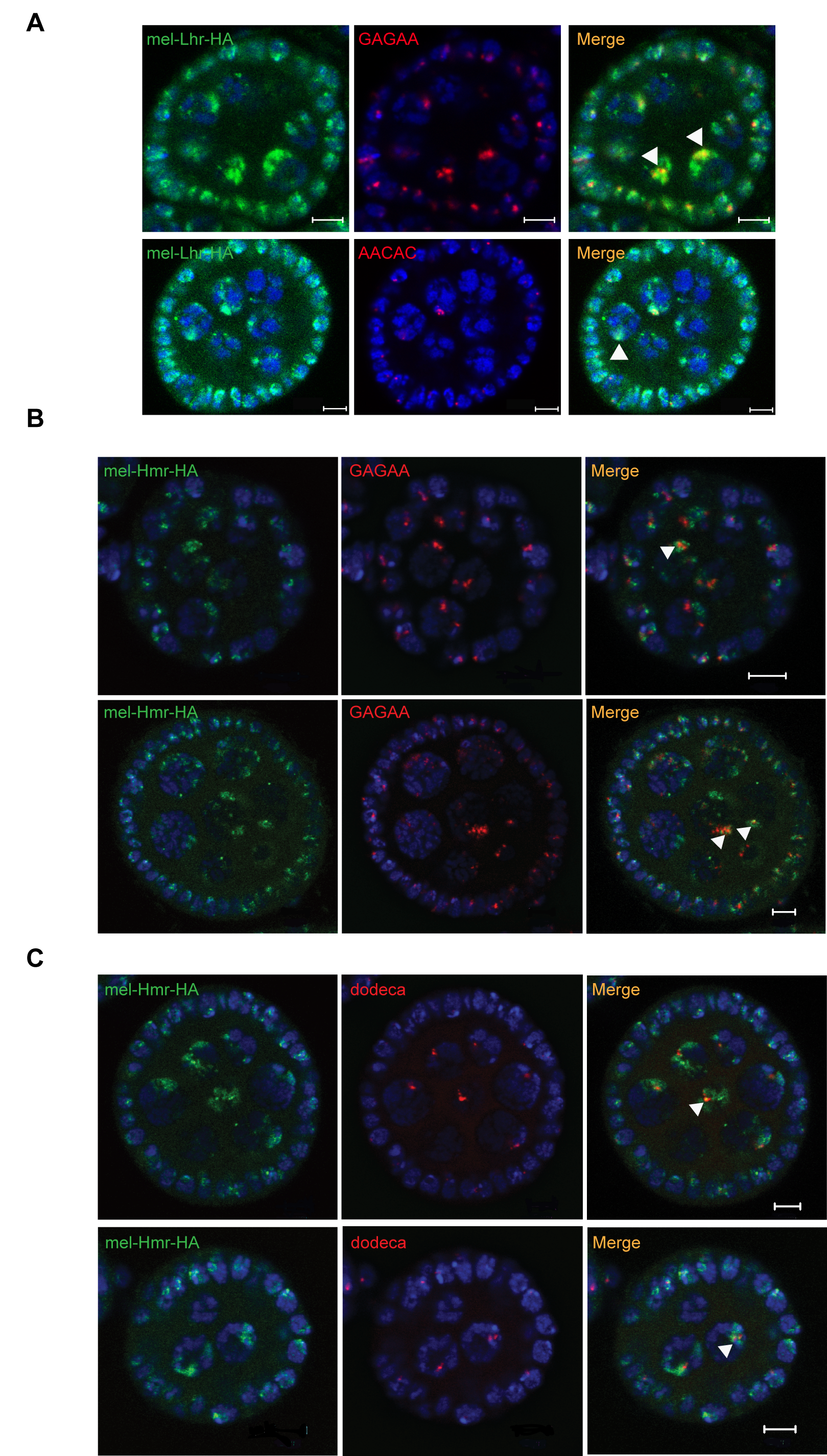

Supplement: Figure S1 — Lhr and Hmr colocalize with specific satellite sequences in ovaries. Nurse cell nuclei (blue) are stained with DAPI in all panels. Scale bars represent 5 µm. (A) mel-Lhr-HA (green) colocalizes with GAGAA(red, top panel) and AACAC (red, bottom panel) in the nurse cells of LhrKO/+; LhrHA/+ ovaries. Arrows point to overlaps between bright FISH and HA-staining foci. (B) mel-Hmr-HA (green) colocalizes with GAGAA (red) and (C) dodeca (red) in nurse cells of Hmr3; mel-Hmr-HA/mel-Hmr-HA ovaries in a subset of nuclei. Arrows point to overlaps between FISH signals and the brightly staining foci of mel-Hmr-HA. Two different egg chambers are shown for both dodeca and GAGAA. (TIF) [file pgen.1004240.s001.tif]

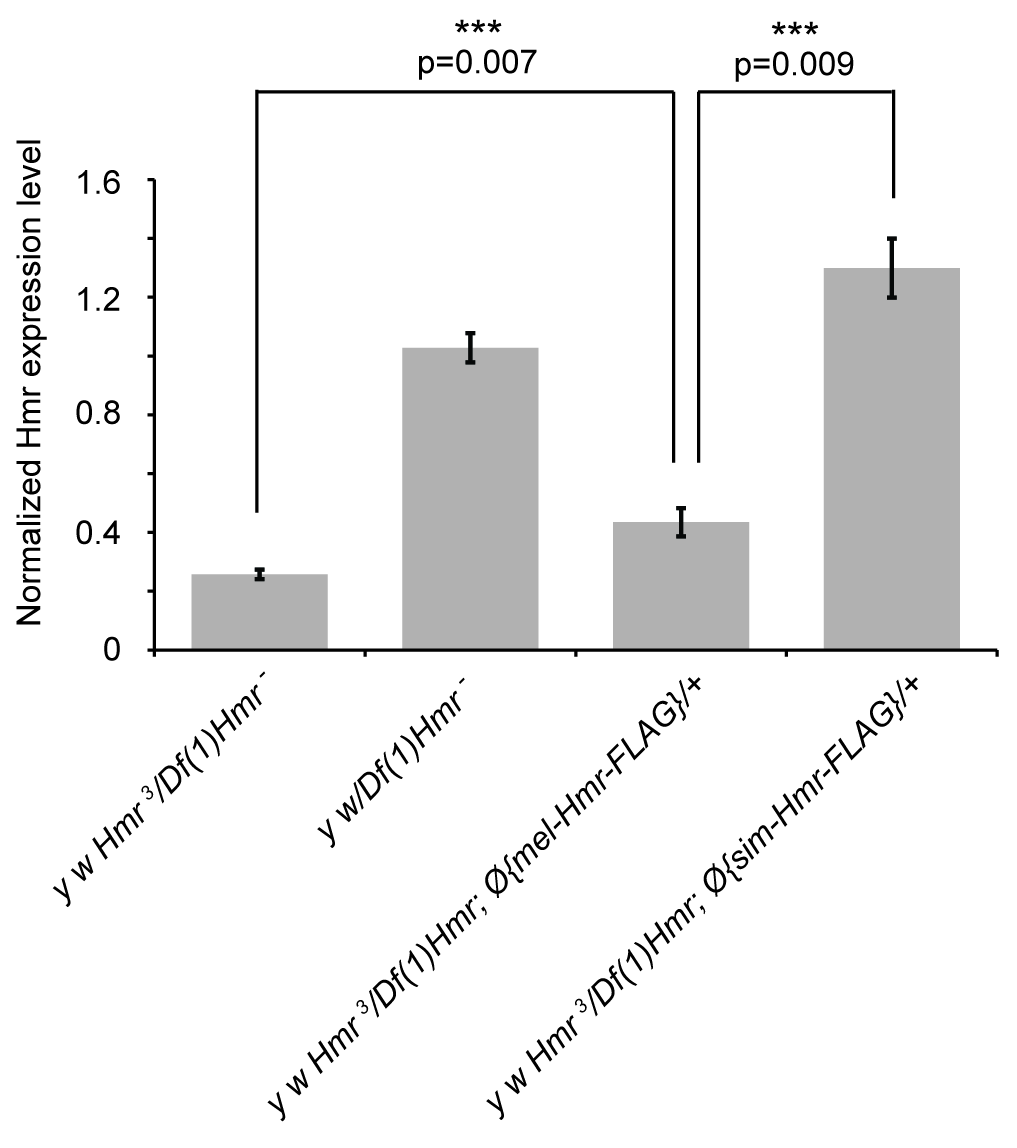

Supplement: Figure S2 — qRT-PCR analysis of Hmr-FLAG transgenes. Hmr transcript levels in transgenic lines were compared to the host strain (Hmr−) and also to Hmr+/ −. The transgenes are heterozygous, therefore both the transgenic lines and Hmr+/− carry one copy of Hmr+. RNA was isolated from ovaries and Hmr expression levels were normalized relative to RpL32. Error bars represent standard error within 3 biological replicates. The difference in the expression level of mel-Hmr-FLAG and sim-Hmr-FLAG is significant (p = 0.009, two-tailed t-test with equal variance). Additionally, the expression of mel-Hmr-FLAG is significantly different than an endogenous copy of Hmr (p = 0.007, two-tailed t-test with equal variance). (TIF) [file pgen.1004240.s002.tif]

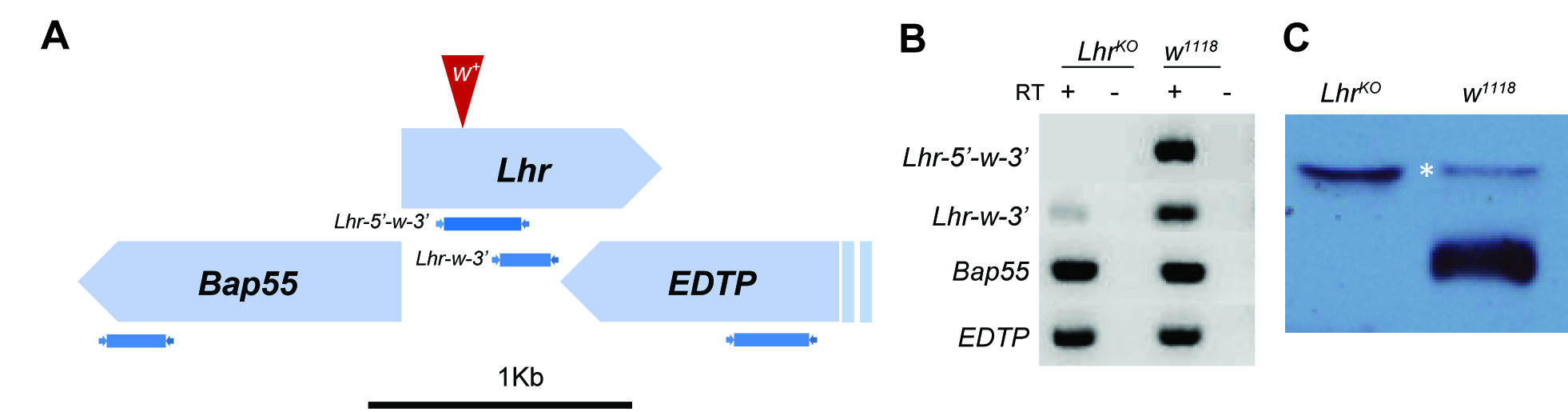

Supplement: Figure S3 — The D. melanogaster LhrKO allele generated by homologous recombination. (A) Lhr and flanking genes are shown, the red triangle labeled w+ indicates the site of the insertion in the LhrKO allele, which is predicted to be ∼4.7 kb based on the structure of the targeting vector. Products used in RT-PCR reactions in (B) are shown below the genes. EDTP gene is partial; w+ insertion not to scale. (B) RT-PCR from adult females shows no Lhr transcript spanning the w+ insertion (Lhr-5′-w-3′) in LhrKO. A highly reduced amount of Lhr transcript is detected 3′ to the w+ insertion (Lhr-w-3′). The flanking genes Bap55 and EDTP are not affected. w1118 was used as a Lhr+ control. +, − indicates presence or absence of reverse transcriptase (RT). (C) Western analysis shows that LhrKO produces no protein. A non-specific band indicated by the asterisk is used a loading control. (TIF) [file pgen.1004240.s003.tif]

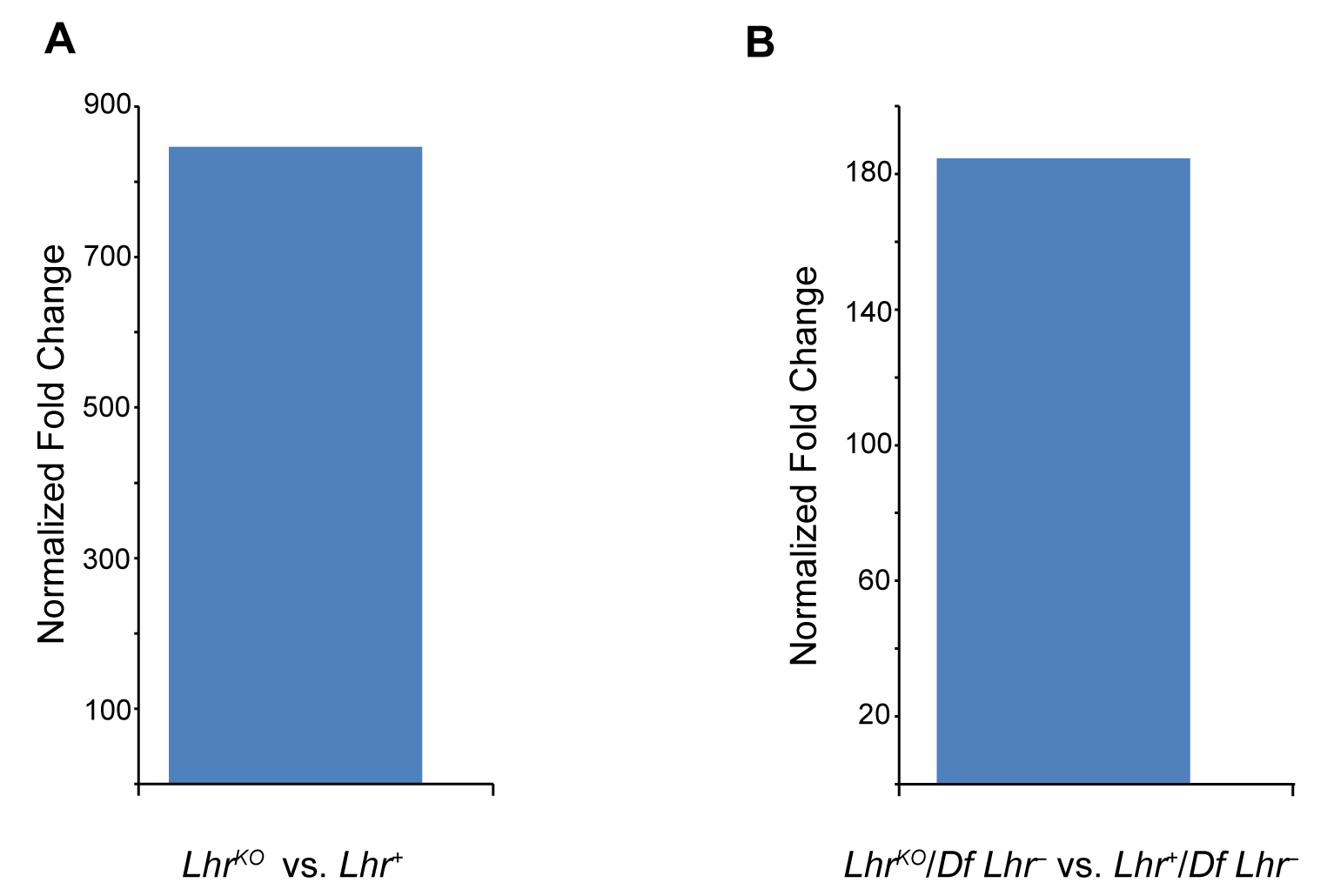

Supplement: Figure S4 — qRT-PCR analysis shows elevated HeT-A levels in Lhr mutants. qPCR was used to estimate the transcript levels of HeT-A relative to the gene RpL32 in poly-A primed cDNA samples obtained from ovarian RNA from two different Lhr− backgrounds and matching controls. (A) Ratio of HeT-A/RpL32 in LhrKO vs. Lhr+, showing mean from 3 biological replicates. Significance of fold change was calculated using Welch's one-tailed t-test; p<0.05. (B) Ratio of HeT-A/RpL32 in LhrKO/Df(2R)BSC44 vs. Lhr+/Df(2R)BSC44, showing mean from 4 biological replicates. Significance of fold change was calculated using the one tailed Wilcoxon rank sum test; p<0.05. (TIF) [file pgen.1004240.s004.tif]

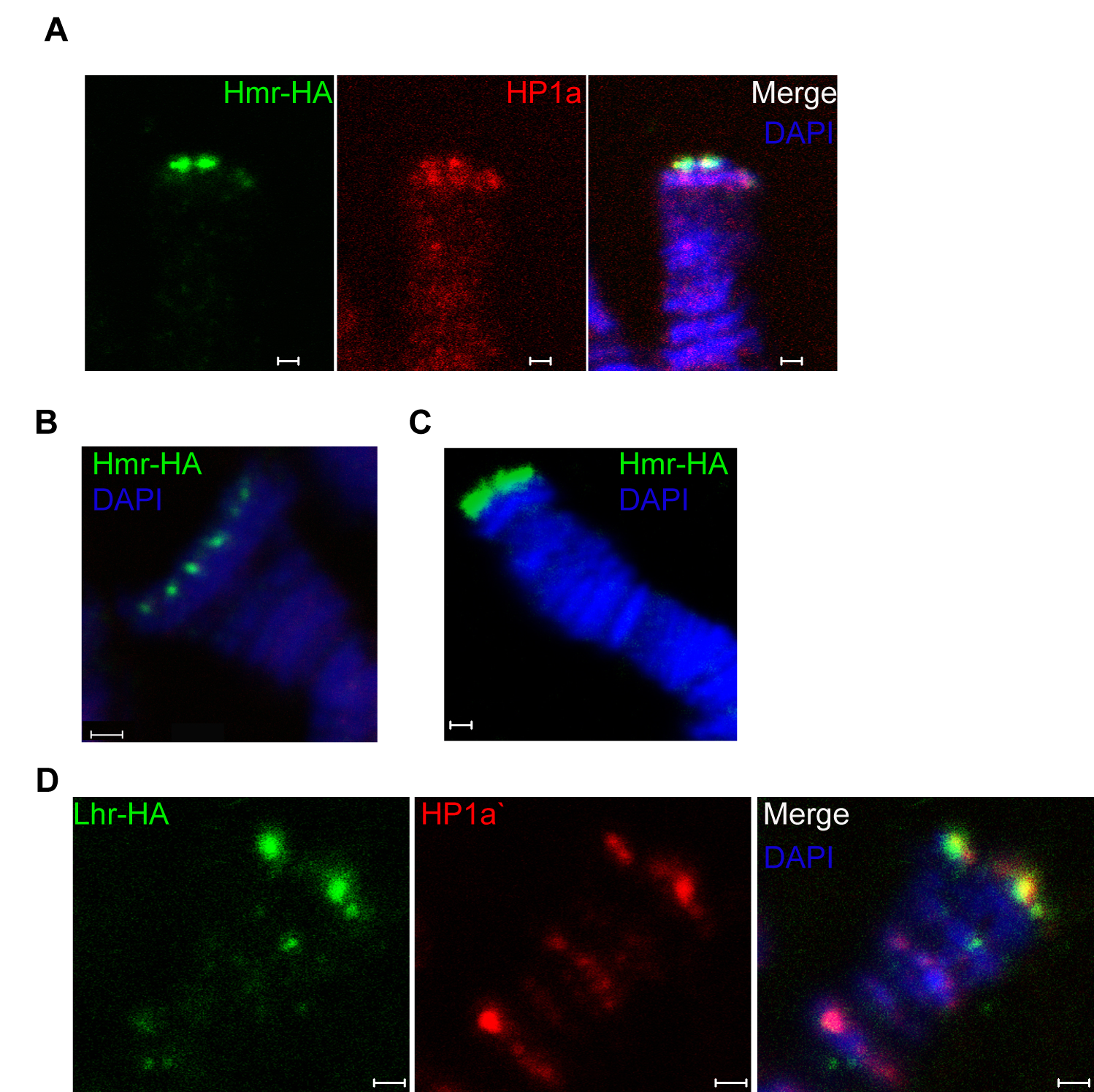

Supplement: Figure S5 — Localization of Hmr-HA and Lhr-HA to the telomeres is independent of dosage of endogenous copies. mel-Hmr-HA (green) in Hmr3; Hmr-HA (A–C) and mel-Lhr-HA (green) in LhrKO/+; Lhr-HA/+ (D) colocalize with HP1A (red) at the telomere cap on polytene chromosomes. mel-Hmr-HA shows a range of distributions at the telomere, including punctate (B) and continuous across the chromosome terminus (C). Scale bar is 1 µm. (TIF) [file pgen.1004240.s005.tif]
