# Supplementary material for: The Hmr and Lhr Hybrid Incompatibility Genes Suppress a Broad Range of Heterochromatic Repeats
Source: PLoS Genet. 2014 Mar 20;10(3):e1004240. doi: 10.1371/journal.pgen.1004240 (PMC3961192; doi:10.1371/journal.pgen.1004240)
Supplement: Table S1 — Eggs laid by LhrKO mothers have a reduced hatch rate. Hatching of eggs laid by LhrKO/+ or homozygous LhrKO mothers crossed to wild-type fathers was followed for 36 hrs after egg lay. For LhrKO/+, 34 eggs from days 2–3, 289 from days 5–6 and 668 eggs from days 10–11 were counted. For LhrKO, 46 eggs from days 2–3, 209 from days 5–6 and 287 eggs from days 10–11 were counted. The significance of the difference in the hatch rates of the eggs laid by LhrKO and LhrKO/+ mothers was calculated by one tailed F.E.T., and was significant at all time points (p<10−4). (DOCX) [file pgen.1004240.s006.docx]

Table S1

Eggs laid by *Lhr^KO^* mothers have a reduced hatch rate.

| Maternal Age | Hatch rate *Lhr^KO^/+* | Hatch rate *Lhr^KO^/Lhr^KO^* |
| --- | --- | --- |
| 2-3 days | 64.70 | 17.39 |
| 5-6 days | 51.90 | 8.13 |
| 10-11 days | 54.64 | 2.78 |
